# New insights into how popular electronic cigarette aerosols and aerosol constituents affect SARS-CoV-2 infection of human bronchial epithelial cells

**DOI:** 10.1038/s41598-023-31592-x

**Published:** 2023-04-10

**Authors:** Rattapol Phandthong, Man Wong, Ann Song, Teresa Martinez, Prue Talbot

**Affiliations:** Department of Molecular, Cell and System Biology, University of California, Riverside, CA 92521 USA

**Keywords:** Cell biology, Diseases, Medical research

## Abstract

The relationship between the use of tobacco products and SARS-CoV-2 infection is poorly understood and controversial. Few studies have examined the effect of electronic cigarettes (ECs) on SARS-CoV-2 infection. We tested the hypothesis that EC fluids and aerosols with nicotine promote SARS-COV-2 infection by increasing viral entry into human respiratory epithelial cells. Responses of BEAS-2B cells to JUUL aerosols or their individual constituents were compared using three exposure platforms: submerged culture, air–liquid-interface (ALI) exposure in a cloud chamber, and ALI exposure in a Cultex system, which produces authentic heated EC aerosols. In general, nicotine and nicotine + propylene glycol/vegetable glycerin aerosols increased ACE2 (angiotensin converting enzyme 2) levels, the SARS-CoV-2 receptor; and increased the activity of TMPRSS2 (transmembrane serine protease 2), an enzyme essential for viral entry. Lentivirus pseudoparticles with spike protein were used to test viral penetration. Exposure to nicotine, EC fluids, or aerosols altered the infection machinery and increased viral entry into cells. While most data were in good agreement across the three exposure platforms, cells were more responsive to treatments when exposed at the ALI in the Cultex system, even though the exposures were brief and intermittent. While both nicotine and JUUL aerosols increased SARS-CoV-2 infection, JUUL significantly decreased the effect of nicotine alone. These data support the idea that vaping can increase the likelihood of contracting COVID-19 and that e-liquid composition may modulate this effect.

## Introduction

COVID-19 (corona virus disease-2019), a serious respiratory illness caused by the SARS-CoV-2 virus (severe acute respiratory syndrome coronavirus 2), has resulted in the death of over 1,000,000 people in the United States and an estimated 15,000,000 people worldwide^1,2^. Because smoking can lead to lung diseases, including cancer and increases in viral infection^3,4^, there has been interest in the relationship between tobacco product/nicotine use and COVID-19 infection, progression, and severity, a relationship that is currently poorly understood and sometimes contradictory. While some patient-derived data indicate that smoking protects against COVID-19^5–7^, most data, including several recent meta-analyses, support the conclusion that smoking is a risk factor for the progression of COVID-19^8–10^ and that patients with a smoking history have a higher probability of developing severe COVID-19 symptoms^11^.

The effects of smoking on COVID-19 have been addressed experimentally using in vitro and animal models. SARS-CoV-2 infection is aided by TMPRSS2 (transmembrane serine protease 2), an enzyme that modifies the viral spike protein, enabling it to fuse with the host cell^12^. ACE2 (angiotensin converting enzyme 2), the receptor for SARS-CoV-2 spike protein, is elevated in biopsies from the respiratory tract of human smokers, consistent with smokers being more susceptible to COVID-19^13–17^. Smokers also have elevated cathepsin B, an enzyme involved in spike processing following infection^13^. In a single cell meta-analysis across various tissues, smoking was correlated with increased levels of ACE2 and TMPRSS2, which was differentially regulated in different respiratory cell types of smokers ^18^. Cigarette smoking also increased SARS-CoV-2 infection of human cells^19,20^, inhibited repair of airway basal cells, and reduced the innate immune system by suppressing interferon β-1^19^, which may contribute to the severity of COVID-19.

Electronic cigarettes (ECs) are nicotine delivery devices that have gained popularity in the last decade^21–23^. ECs heat e-liquids to produce aerosols that contain numerous chemicals, some of which are distinct from those in tobacco smoke^4,24,25^. EC aerosols can have multiple adverse effects on the respiratory system^22,26–29^. These include negative effects on lung physiology and the immune response, which may make fighting and recovery from a COVID-19 infection more difficult.

The effects of vaping on SARS-CoV-2 infection have been examined in humans and mice and in vitro with human cells. The bronchial-lavage fluid from EC users and smokers had elevated ACE2 activities^20^. Full body exposures to “Mint”, but not “Mango”, aerosols from JUUL pods increased ACE2 levels in mouse lung^30^. Aerosols containing a mixture of PG (propylene glycol)/VG (vegetable glycerol) and nicotine elevated ACE2 levels^31–33^. While these studies reported gender differences, they were not in agreement with respect to the gender affected.

The effects of ECs on ACE2 levels have been studied in vitro with cultured human cells from the respiratory system. JUUL fluids increased ACE2 enzymatic activity in vitro^20^. Although ACE2 serves as a spike protein receptor, its enzymatic activity is not required for infection^34^. PG/VG elevated ACE2 mRNA expression in BEAS-2B cells treated with e-liquids in submerged culture, and the effect was dependent on flavor chemicals and nicotine in the e-liquid mixture^35^. JUUL “Virginia Tobacco” e-liquid increased infection in primary tracheobronchial and small airway epithelium in submerged culture^20^; however, e-liquid rather than aerosol was tested.

The relationship between vaping EC aerosols and the acquisition of COVID-19 is poorly understood. To add clarity to this topic, we tested the hypothesis that EC aerosols increase the levels of ACE2 and the levels and activities of TMPRSS2 in human bronchial epithelium, leading to an increase in viral infection. We performed controlled laboratory experiments with human cells that were exposed to known concentrations of both JUUL “Virginia Tobacco” EC fluids and aerosols, nicotine, PG and VG, and then examined discrete endpoints relevant to infection. We chose JUUL “Virginia Tobacco” because this product is currently marketed, popular, and has few flavor chemicals^36^, making its fluid and aerosol a relatively simple mixture for testing. Moreover, tobacco flavored products are likely to remain marketable in the future as they are not subject to the FDA’s enforcement policy that proposes to remove flavored EC products from the market^37^. We tested both JUUL “Virginia Tobacco” fluid and aerosols equivalent to those inhaled during vaping. Testing was done across three in vitro exposure platforms: submerged cultures, cloud chamber ALI exposure, and Cultex ALI exposure. Submerged culture has historical importance and is still frequently used^38–42^. The cloud chamber enables individual chemicals to be studied without using heat to create the aerosol, so that endpoint effects can be attributed to a particular chemical(s) without interference from other chemicals, solvents, or reaction products ^43^. The Cultex system was used to vape JUUL ECs by heating their fluid to create JUUL aerosols, which are equivalent to those inhaled by EC users. Cultex-generated aerosols contain the chemicals normally present in the fluid^25^ plus any reaction products or metals added when fluid is heated in an EC atomizer^44–50^. Infection of cells was evaluated using SARS-CoV-2 pseudoparticles with a green-fluorescent reporter protein. Data were compared across exposure platforms and across exposure groups (JUUL fluids, JUUL aerosols, and individual chemicals).

## Materials and methods

### BEAS-2B, HEK 293T, and HEK 293T^***ACE2***^ cell culture and maintenance

BEAS-2B cells (ATCC, Manassas, VA, USA) were cultured in Bronchial Epithelial Basal Medium (BEBM; Lonza, Walkersville, MD, USA) supplemented with the Bronchial Epithelial Growth Bullet Kit (BEGM; Lonza, Walkersville, MD, USA) without the gentamycin antibiotic. Cells were cultured in Nunc T-25 tissue culture flasks (Fisher Scientific, Tustin, CA, USA), pre-coated overnight with collagen type I, bovine serum albumin, and fibronectin. Cells were passaged at 80% confluency. Cell cultures were washed with Dulbecco’s phosphate buffered saline without calcium or magnesium (DPBS-; Lonza, Walkersville, MD, USA) and detached with 0.25% trypsin with 0.53 mM EDTA (ATCC, Manassas, VA, USA) and poly-vinyl-pyrrolidone (Sigma-Aldrich, St. Louis, MO, USA). Cells were seeded at 3,000 cells/cm^2^ in a pre-coated T-25 flask, and culture medium was replaced every other day.

For in vitro submerged treatments, cells were seeded at a density of 9,000 cells/cm^2^ in pre-coated 6-well and 12-well plates and allowed to attach overnight prior to treatments. For ALI culture, cells were seeded at a density of 12,000 cells/cm^2^ in pre-coated 12-well Transwell inserts with a pore size of 0.4 µm (Corning, Inc., Corning, NY, USA) and allowed to form a monolayer. Once a monolayer formed, the medium on the apical side of the transwell was removed, and the monolayer was acclimated to air for 24 h prior to an exposure. Medium in the basal-lateral side of the transwell was replaced every other day. Cells were incubated at 37 °C, 5% CO_2_, and 95% relative humidity.

HEK 293T cells (ATCC, Manassas, VA, USA), and HEK 293T cells over expressing ACE2 (HEK 293T^ACE2^, BEI resource, Manassas, VA, USA; NR-52511) were cultured in DMEM high glucose medium supplemented with 10% fetal bovine serum (FBS; ATCC Manassas, VA, USA). At 80–90% confluency, cells were washed and detached as described above. Cells were seeded at 3,000 cells/cm^2^ and incubated in a 37 °C, 5% CO_2_, 95% relative humidity incubator.

### Submerged treatments

Refill fluids contained PG (Thermofisher, Tustin CA, USA) and VG (Thermofisher, Tustin CA, USA). At 80% confluency, BEAS-2B cells were treated for 24 h with nicotine, PG/VG, PV/VG with nicotine, or JUUL fluids. All treatments were diluted with BEGM culture medium to reach working concentrations. Liquid (-)- nicotine (Sigma-Aldrich, St. Louis, MO, USA) was diluted to reach final concentrations of 0.03 mg/mL or 0.3 mg/mL. PG/VG (30/70 ratio) was diluted to reach a final concentration of 0.5% in volume. Stock solutions of PG/VG with nicotine were made and then diluted with culture medium to 0.5% PG/VG with either 0.03 mg/mL nicotine or 0.3 mg/mL nicotine. JUUL “Virginia Tobacco” was diluted to 0.5%, which contains PG/VG at 0.5% concentration and nicotine at 0.3 mg/mL.

### Nicotine aerosol exposure at the ALI in the VITROCELL cloud chamber

Monolayers of BEAS-2B cells cultured on 12 well Transwell inserts were placed into a VITROCELL cloud chamber (VITROCELL Walkirch, Germany) for an ALI exposure of various chemical aerosols that were generated without heating. Prior to exposures, pre-warmed culture medium was dispensed into wells of the exposure chamber and allowed to equilibrate to 37 °C. Stock nicotine was diluted with PBS- to make exposure solutions with final concentrations of either 0.03 mg/mL or 0.3 mg/mL nicotine. For each exposure, 200 µL of exposure solution was added into a VITROCELL nebulizer to generate a uniform aerosol with a flow rate of 200 µL/min without using heat. Control cells were exposed to PBS- aerosols.

BEAS-2B cells cultured on 12-well inserts were exposed to 1 puff of PBS- and nicotine (0.03 or 0.3 mg/mL) aerosol during a 1.5-min aerosol generation period followed by a 3-min aerosol deposition period. After the exposure, the cells were returned to the incubator to recover for 24 h.

### EC Aerosol exposure at the ALI in the Cultex RFS compact exposure system

A Cultex RFS compact exposure module (Cultex Laboratories GmbH, Hannover, Germany) was used to expose cells to humified sterile air (clean air control) or EC aerosols generated from EC devices. Prior to each exposure, cells were placed into the exposure chambers, which contained culture medium maintained at 37 °C.

The Cultex exposure system consisted of a sampling module and an aerosol guiding module. The sampling module contained a custom designed EC smoking robot (RTI International, North Carolina, USA) that draws filtered air from the biosafety cabinet or EC aerosol from the EC device into a 200 mL syringe. After collecting a 55 mL sample of either filtered air or EC aerosols, the Cultex system then dispensed the sample into the aerosol guiding module, where it was combined with humified zero air (1 L/min). This mixture step diluted the sample and generated a uniform flow before the exposure mixtures were directly distributed onto each biological sample. Exposure mixtures were allowed to settle onto cells for 5 s, then vented out of the exposure chamber at a flow rate of 5 mL/min, generated by a mass flow controller (Boekhorst, Bethlehem, PA, USA), and finally dispensed into a waste container.

Each exposure consisted of 55 mL of filtered air or EC aerosol generated using a 4 s puff duration and a 30 s puff interval. BEAS-2B cells were exposed to 10 puffs of either clean air or EC aerosol and allowed to recover for 24 h prior to analyses.

### Quantification of deposited nicotine in the ALI exposure systems

To quantify the nicotine deposited in the ALI exposure systems, exposures were repeated as described in the previous protocol sections using isopropyl alcohol (IPA; Fisher Scientific, Fair Lawn, NJ, USA) in place of the biological samples. Ten mL of IPA were added to each exposure well in both the cloud chamber and Cultex system. After the exposures, the IPA was collected and shipped overnight on ice to Portland State University where it was analyzed using gas chromatography-mass spectrometry (GC–MS). Chemical analysis was performed with an Agilent 5975 GC/MS system (Agilent, Santa Clara, CA, USA) using an internal standard-based calibration procedure and method previously described^25,51^.

### Immunocytochemistry of BEAS-2B cells

BEAS-2B cells were seeded in 8-well chamber slides (Ibidi, Gräfelfing, Germany). At 80% confluency, cells were treated with nicotine or EC fluids for 24 h in an incubator. Cells were then fixed in 4% paraformaldehyde for 15 min at room temperature and washed several times with DPBS + . Samples were permeabilized with 0.1% Triton X and blocked using 10% donkey serum (Sigma-Aldrich, St. Louis, MO, USA) for 1 h at room temperature, followed by an overnight incubation in primary antibody. After several washes with PBS-T (DPBS + 0.1% Tween), the samples were incubated at room temperature in the dark for 2 h with appropriate secondary antibodies. Samples were washed several times and mounted using Vectashield with DAPI (Vectashield, San Francisco, CA, USA). Fluorescent cells were imaged with a Nikon Eclipse Ti inverted microscope (Nikon Instrument, Melville, NY, USA) using a 60X objective, and images were captured using a high-resolution Andor Zyla VSC-04941 camera (Andor, Belfast, UK). Antibodies used were anti-ACE2 (1:100; R&D System, Minneapolis MN, USA), anti-TMPRSS2 (1:200; Santa Cruz, Dallas, TX, USA). Secondary antibodies were Alexa fluor-488 or Alexa fluor-594 (Thermofisher, Tustin CA, USA).

### Lysate preparation for western blot and proteolytic assay

After submerged treatments or exposures at the ALI, RIPA buffer with or without PMSF protease inhibitors (ChemCruz Biochemical, Dallas, TX, USA) was used to lyse cells. Lysates for Western blots were prepared using RIPA buffer with protease inhibitors, while lysates for proteolytic assays used RIPA buffer without protease inhibitors. The cell lysates were vortexed every 10 min for 30 min, pipetted through 23-gauge needles several times, then centrifuged at 3,000 × g for 5 min at 4 °C. The lysate samples were separated from insoluble pellets. The lysate protein was quantified using the Pierce BCA assay kit (Thermo Scientific, Waltham, MA. USA). Each Western blot used 30 µg of protein, and each proteolytic assay used 5 µg of protein.

### Western blotting

Following lysate preparation, denaturing buffer (β-mercaptoethanol and Laemeli buffer, 1:10) was added to each Western blot lysate at a 1:4 ratio. The buffer/lysate mixtures were heated at 95 °C for 2 min, then loaded onto an any kD Mini-PROTEAN TGX precast protein gel (BioRad, Carlsbad, CA, USA) for electrophoretic separation of proteins (120 V for 1–2 h), and afterwards transferred to a BioRad PVDF membrane at 200 mA overnight at 4 °C. Following transfer, the membrane was cut horizontally, either below or above the expected location of the protein of interest based on its molecular weight (kDa). The membranes were then blocked with 5% milk in TBS-T (TBS with 1%Tween-20) buffer for 2 h and incubated overnight at 4 °C with antibodies against ACE2 (1:300; R&D systems, Minneapolis, MN, USA), TMPRSS2 (1:1000; Santa Cruz, Dallas, TX, USA), and GAPDH (1:2000; Cell Signaling Technology, Danvers, MA, USA). Next, the membranes were washed for 30 min in TBS-T, then incubated in an HRP-conjugated secondary antibody (1:1000; Santa Cruz, Dallas, TX, USA or Cell Signaling Technology, Danvers, MA, USA) for 2 h at room temperature. Finally, the membranes were developed using BioRad Clarity Western ECL Substrate reagent (BioRad, Carlsbad, CA, USA) in a BioRad ChemiDoc Imaging System (BioRad, Carlsbad, CA, USA), which determined the optimal exposure time for each protein (ACE2 = 120 ms; TMPRSS2 = 24 ms; GAPDH = 4 ms).

### TMPRSS2 proteolytic assay

The TMPRSS2 enzyme assay was performed using a modification of a previously published protocol^52^. The fluorogenic substrate, Boc-Gln-Ala-Arg-AMC · HCl, (Bachem, Torrance, CA. USA) was dissolved in DMSO and diluted in reaction buffer (50 mM Tris pH 8, 150 mM NaCl) to a final concentration of 10 µM. The fluorogenic substrate was added to each well of a 96-well plate and the fluorescence intensity was measured at 340/440 nm over a 1 h period at 37 °C using a Bio-Tek Synergy HTX (Agilent, Santa Clara, CA, USA) plate reader.

### Spike viral pseudoparticle production

Pseudoparticle production was performed as outlined in Crawford et al.^53^. In brief, HEK293T cells were plated with antibiotic-free medium at a density of 7 × 10^6^/T75 flask and transfected with Lipofectamine3000 (Thermo Fisher Sci, Waltham, MA. USA) using a total of 15 µg of BEI (BEI resources, Manassas, VA, USA) lentiviral plasmids (NR-52520: pHAGE2-CMV-ZsGreen-W, 7.5 µg; NR-52517: HDM-Hgpm2, 1.65 µg; NR-52519: pRC-CMV Rev1b, 1.65 µg; NR-52518: HDM-tat1b, 1.65 µg; NR-52314: pHDM-SARS-CoV-2 Spike, 2.55 µg), 60 µL of Lipofectamine, and 20 µL of P3000 reagent following the manufacturer's protocol. After overnight incubation, fresh medium supplemented with 1% bovine serum albumin (BSA; Sigma-Aldrich, St. Louis, MO, USA) was added to the cells. Fluorescence microscopy was used to visually inspect transfection efficiency and the expression of ZsGreen**.** Wild-type HEK293T cells, which were not transfected by the lentiviral plasmids, were used as a transfection control. The cell culture medium from transfected and wild-type HEK293T cells was collected 48 h after transfection, centrifuged, and the resulting supernatant was filtered using a 0.45 µm syringe filter. The filtered supernatant was mixed with 5X PEG (Abcam, Cambridge, UK) and precipitated overnight at 4 °C. The lentivirus was collected by centrifugation and resuspension of the pellet in Viral Re-suspension Solution (Abcam, Cambridge, UK). The virus aliquots were stored at − 80 °C. Prior to infection experiments, the transduction efficiency of each viral batch was determined by infecting 293T^ACE2^ cells and quantifying the number of infected cells with flow cytometry. The medium from transfection control cells were processed the same, and produced a mock transfection solution, which did not contain virus and was used for mock infection.

### Viral pseudoparticle infection

For each viral pseudoparticle infection experiment, a 0.5 multiplicity of infection (MOI) was used to infect BEAS-2B cells. Viral pseudoparticles were delivered as a mixture with the appropriate fresh culture medium.

In the submerged culture infection experiments, cells were treated for 24 h, then the treatment was replaced with pseudoparticle medium. In the ALI culture infection experiments, following the recovery period, 100 µL of pseudoparticle medium was added directly onto the apical side of the Transwell.

In all infection experiments, cells or tissues were incubated with viral pseudoparticles for 24 h. Subsequently, the pseudoparticles were removed and the cells or tissues were allowed to incubate another 24 h to amplify the expression of the green fluorescence reporter protein in the infected cells. Cells were harvested and analyzed with flow cytometry to determine the number of infected cells.

### Flow cytometry

Prior to flow cytometry (Novocyte, Agilent Technologies, Santa Clara, CA, USA), fluorescence microscopy was used to validate the expression of ZsGreen signal. All samples were pipetted several times and passed through a 35 µm filter of a Falcon Round-Bottom 5 mL polystyrene test tube (Fisher Scientific, Tustin, CA, USA) to generate single cell suspensions. Forward-Scatter-Height (FSC-H) and Forward-Scatter-Area (FSC-A) were used to generate a gate to select single cells for each sample. A gate to exclude small debris was created using Side-Scatter-Area (SSC-A) and FSC-A. Non-fluorescent mock infected cells, which were incubated in viral re-suspension solution without viral pseudoparticles, were used to produce a gate to quantify the fluorescence signal of infected cells. Final results were represented as percent of fluorescent infected cells in each sample.

### Statistical analysis

In all cases, three independent experiments were performed using different passages of BEAS-2B cells. Statistical analyses were done using Minitab Statistics Software (Minitab, State College, PA, USA). Data were first checked for normality of distribution and homogeneity of variances. When data did not satisfy the assumptions of analysis of variance (ANOVA), they were subjected to a Box-Cox transformation, and again checked to verify that data satisfied the ANOVA model. Statistical analyses were done using one-way ANOVA. When significance was found (*p* < 0.05), data were further analyzed using Tukey’s multiple comparison posthoc test to isolate significant differences to specific groups. Means were considered significantly different for *p* < 0.05. Data were plotted using GraphPad Prism 7 software (GraphPad, San Diego, CA, USA).

## Results

### Effect of JUUL fluids, aerosols, and EC chemicals (nicotine and PG/VG) on ACE2 in BEAS-2B cells during submerged culture and ALI exposures

**Figure 1:**
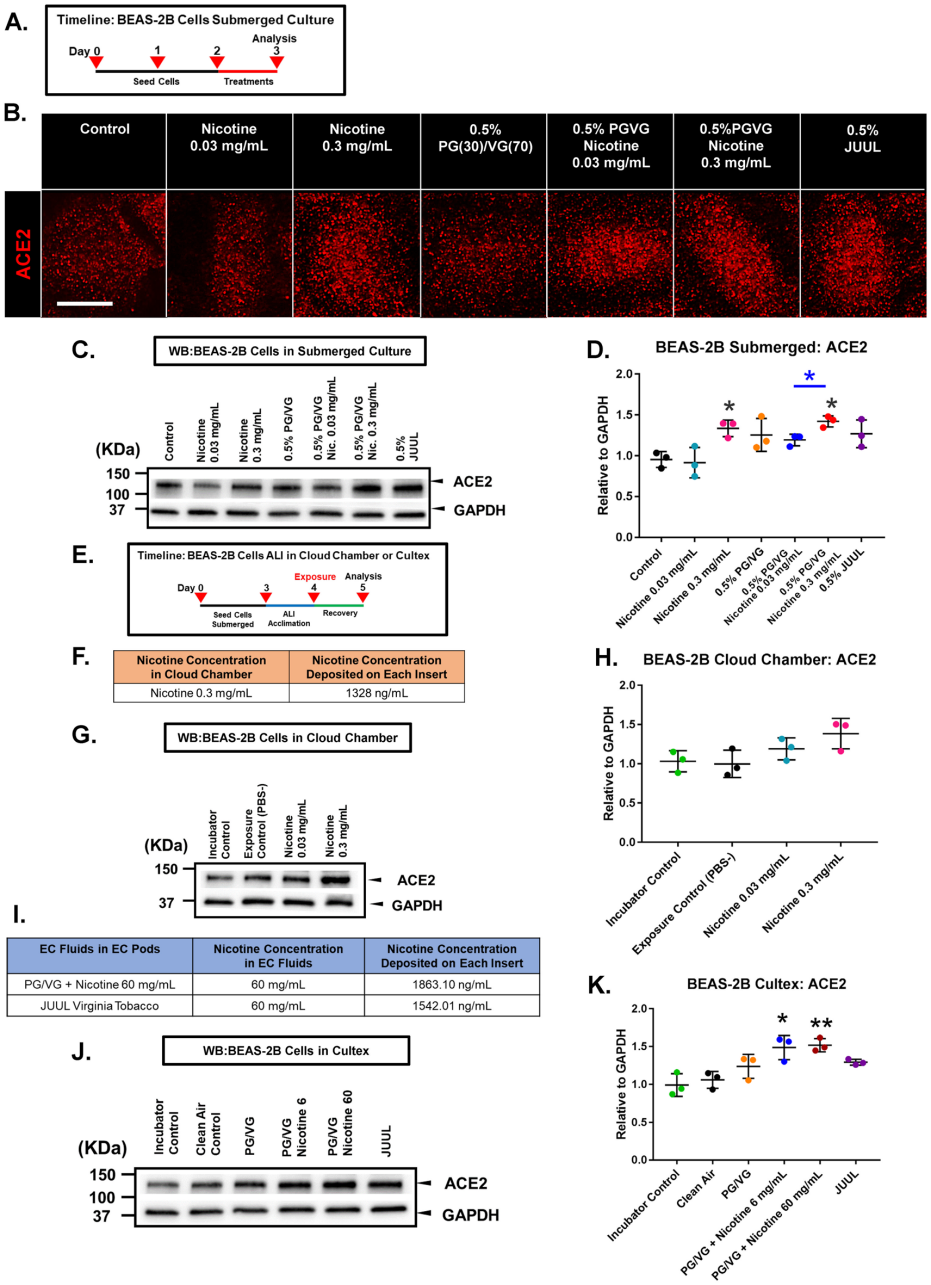
ACE2 expression in BEAS-2B cells during exposure in submerged culture and at the air–liquid interface. (**A**) Experimental treatment used in submerged culture. (**B**) Micrographs showing cells labeled with ACE2 antibody. Scale bar = 15 µm. (**C–D**) The effect of JUUL e-liquid, nicotine, and PG/VG on ACE2 during submerged culture (Western blot). (**E**) Experimental treatment used with cells exposed at the ALI in a cloud chamber or the Cultex system. (**F**) Concentration of nicotine deposited in each well following aerosolization of 0.3 mg/mL of nicotine in a cloud chamber. (**G**–**H**) The effect of 1 puff of nicotine-containing aerosol on ACE2 following ALI exposure in a cloud chamber (Western blot). (**I**) Nicotine concentration in each well following exposure to aerosol generated from PG/VG/nicotine 60 mg/mL or JUUL “Virginia Tobacco” in a Cultex system. (**J**–**K**) The effect of 10 puffs of nicotine-containing EC aerosols on ACE2 following ALI exposure in a Cultex system. The aerosols were generated from PG/VG, PG/VG with nicotine, and a JUUL EC. Data are plotted as the mean ± standard deviation of three independent experiments. Box-Cox transformed data were analyzed using a one-way ANOVA followed by Tukey’s test to compare means. Black * = significantly different than the control. Colored * and colored lines = significantly different groups. *p < 0.05, **p < 0.01. Western blots were horizontally cropped from the original blot.

The effects of JUUL “Virginia Tobacco” fluids and aerosols and individual components in JUUL products (specifically nicotine and PG/VG) on ACE2 levels were examined in BEAS-2B cells using submerged and ALI exposures (Fig. 1). The experimental timeline for the submerged exposure experiment is shown in Fig. 1A. BEAS-2B cells were treated in submerged culture for 24 h with 0.5% (0.03 mg/mL) or 5% (0.3 mg/mL) nicotine JUUL “Virginia Tobacco” fluid, pure nicotine (0.03 mg/mL or 0.3 mg/mL), 0.5% PG/VG (ratio = 30/70), or 0.5% PG/VG with nicotine (0.03 mg/mL or 0.3 mg/mL). Micrographs of treated BEAS-2B cells labeled with ACE2 antibodies showed increased expression of ACE2 in those groups that contained 0.3 mg/mL of nicotine or PG/VG + nicotine (Fig. 1B). Increased expression was confirmed using Western blots (Fig. 1C–D). Nicotine increased ACE2 significantly in those groups that contained only 0.3 mg/mL of nicotine or only PG/VG + 0.3 mg/mL of nicotine. Even though JUUL “Virginia Tobacco” has 0.3 mg/mL of nicotine, ACE2 in the JUUL treated group was not significantly different than the control. Overall, nicotine (0.3 mg/mL) treatment for 24 h in submerged cultures increased ACE2 levels, while PG/VG alone did not significantly affect ACE2.

In similar experiments, BEAS-2B cells were exposed to aerosols at the ALI in a VITROCELL cloud chamber or Cultex exposure system (Fig. 1E–K). In the cloud chamber, BEAS-2B cells were exposed to either 1 puff of aerosol generated from solutions of PBS or nicotine (0.03 mg/mL or 0.3 mg/mL) in PBS, then allowed to recover for 24 h before Western blotting (Fig. 1G–H). Although ACE2 increased in both nicotine-treated groups, it was not significantly different than the untreated control (Fig. 1H). There was no significant difference between the incubator and PBS controls, indicating that incubation in the cloud chamber did not affect the cells.

In the Cultex system, BEAS-2B cells were exposed to either 10 puffs of humidified, filtered clean air or 10 puffs of aerosols made using JUUL “Virginia Tobacco” ECs. In addition, the JUUL EC battery was used with refillable third-party pods containing lab made refill fluids to determine which chemicals in JUUL fluid affected ACE2 levels. The fillable pods contained PG/VG, PG/VG with 6 mg/mL of nicotine, or PG/VG with 60 mg/mL of nicotine. There was no significant difference between the incubator and clean air controls. The small increase in ACE2 in the PG/VG and JUUL “Virginia Tobacco” aerosols was not significantly different than the clean air control (Fig. 1J and K). Cultex aerosols from lab made fluids containing only PG/VG or PG/VG + nicotine increased ACE2 expression significantly compared to the clean air control.

In both submerged and ALI exposures, nicotine and EC aerosols with nicotine elevated ACE2 levels in BEAS-2B cells.

The exposures in the cloud chamber and Cultex experiments were compared by measuring nicotine deposited in the transwell culture medium after exposure. When aerosolized in the cloud chamber, solutions containing 0.3 mg/mL of nicotine deposited 1,328 ng/mL of nicotine into the fluid in each insert (Fig. 1F). In the Cultex system, 1,863 ng/mL of nicotine were deposited into each insert from aerosols generated using PG/VG with 60 mg/mL of nicotine. JUUL aerosols deposited a similar concentration of nicotine into the fluid in each insert (1,543 ng/mL) (Fig. 1I). These results demonstrate that similar amounts of nicotine were deposited in each insert in the two ALI exposure systems.

### The effect of JUUL fluids, PG/VG, and nicotine on the concentrations and activities of TMPRSS2 in BEAS-2B cells during submerged exposure

**Figure 2:**
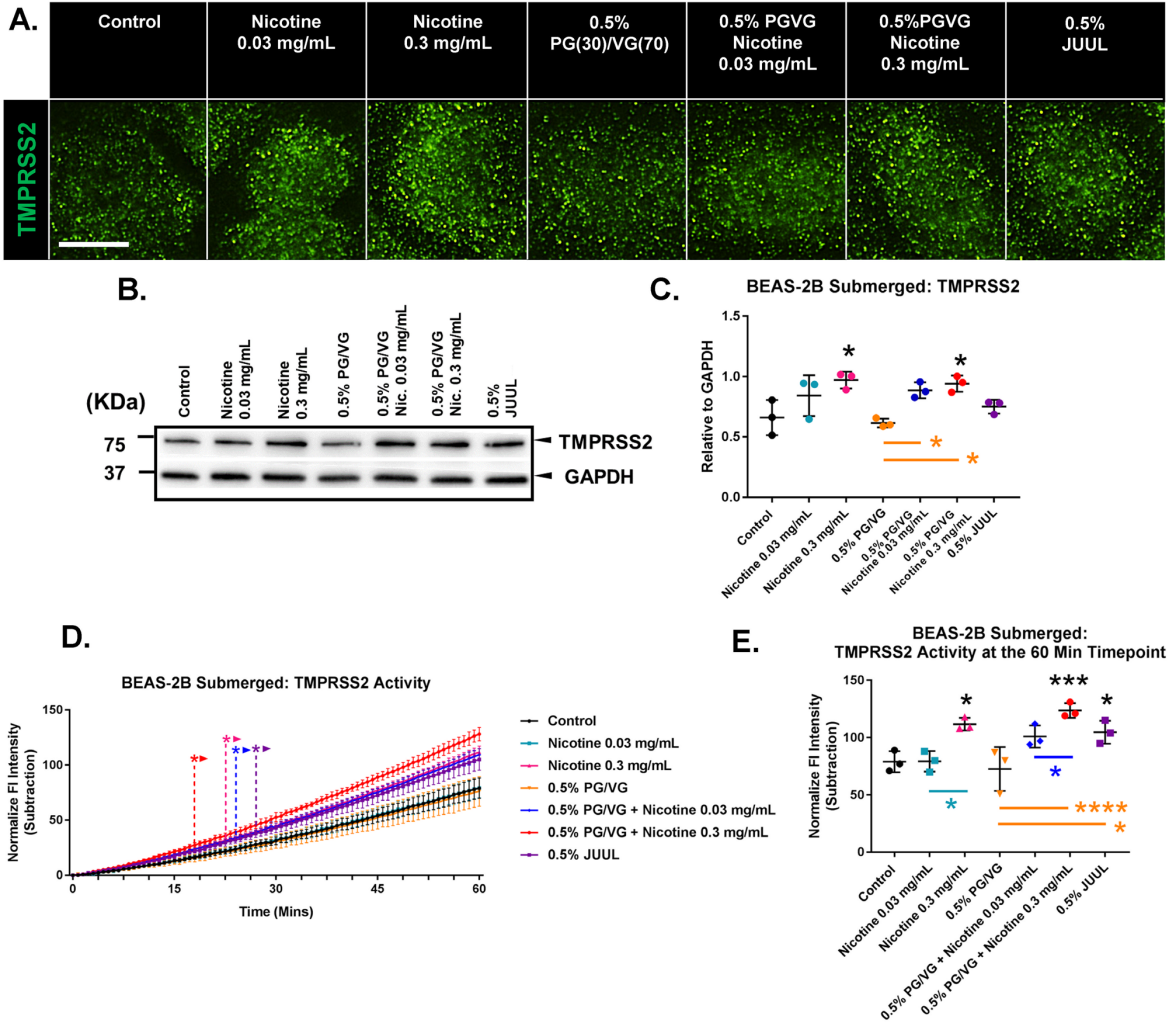
Effect of Juul e-liquid, nicotine, and PG/VG on TMPRSS2 in BEAS-2B cells in submerged culture. (**A**) Micrographs showing cells labeled with TMPRSS2 antibody. Scale bar = 15 µm. (**B**–**C**) Effect of JUUL e-liquid, nicotine, and PG/VG on TMPRSS2 levels in submerged culture (Western blot). (**D**–**E**) TMPRSS2 activity was increased by JUUL e-liquid and nicotine following 24 h of treatment in submerged cultures. (**D**) Changes in activity over 60 min. * Indicate when values became significantly different than the control (*p* ranged from 0.05 to 0.0001). (**D**) was analyzed using a two-way ANOVA followed by Dunnett’s compared to the control. (**E**) Activity at the 60-min timepoint. All graphs show the mean ± standard deviation of three independent experiments. In **C** and **E** Box-Cox transformed data were analyzed using a one-way ANOVA followed by Tukey’s test. Black * = significantly different than the control. Colored * and lines = significantly different groups. *p < 0.05, **p < 0.01, ***p < 0.001, ****p < 0.0001. Western blots were horizontally cropped from the original blot.

Submerged treatments were done to determine the effects of PG/VG, nicotine, and JUUL “Virginia Tobacco” fluid on TMPRSS2 levels and enzymatic activity (Fig. 2). TMPRSS2 is critical for infection, as it modifies SARS-CoV-2 spike protein after binding to ACE2 and facilitates virus and host membrane fusion.

Similar exposure experiments were done to determine if TMPRSS2 is affected by JUUL “Virginia Tobacco” fluid, PG/VG, or nicotine. Treated BEAS-2B cells labeled with TMPRSS2 antibodies showed increased expression of TMPRSS2 in those groups exposed to nicotine (Fig. 2A). In Western blots, TMPRSS2 expression was not significantly affected by JUUL fluid or PG/VG alone (Fig. 2B, C). However, groups treated with lab-made fluids containing 0.3 mg/mL of nicotine had elevated levels of TMPRSS2 (Fig. 2B, C). TMPRSS2 activity, as measured by cleavage of a specific fluorescent substrate, significantly increased when BEAS-2B cells were exposed to JUUL “Virginia Tobacco” EC fluid, 0.3 mg/mL of nicotine, or 0.3 mg/mL of nicotine + 0.5% PG/VG (Fig. 2D, E). PG/VG alone did not significantly affect TMPRSS2 activity.

### The effect of JUUL, PG/VG, and nicotine aerosols on TMPRSS2 concentration and activity in BEAS-2B cells following ALI exposures

Similar experiments were performed with BEAS-2B cells exposed at the ALI to aerosols generated in a cloud chamber or Cultex exposure system. TMPRSS2 levels were evaluated in Western blots, and its activity was analyzed using specific fluorogenic substrates.

**Figure 3:**
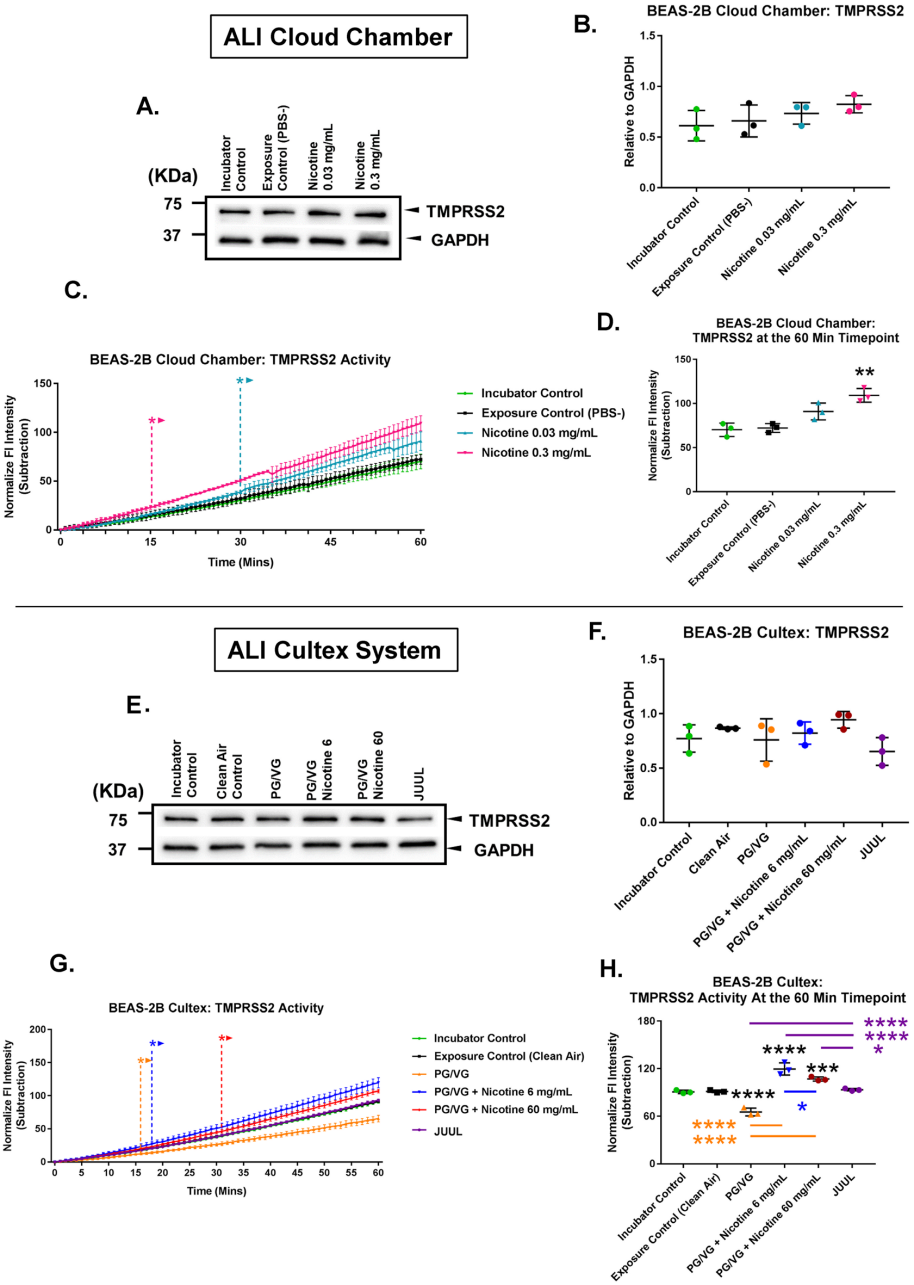
Effect of EC aerosols on TMPRSS2 levels and activity in BEAS-2B cells exposed at the air–liquid interface. (**A, B**) Effect of 1 puff of pure nicotine aerosol on TMPRSS2 levels; exposure was at the ALI in a cloud chamber (Western blot). (**C, D**) TMPRSS2 activity was increased by nicotine following exposure at the ALI in a cloud chamber. (**C**) Changes in activity over 60 min. * Indicate when values became significantly different than the control (*p* ranged from 0.05 to 0.0001). (**D**) Activity at the 60-min timepoint. (**E, F**) Western blot showing the effect of 10 puffs of PG/VG, PG/VG/nicotine, and JUUL EC aerosol on TMPRSS2 levels during ALI exposure in the Cultex system. (**G, H**) Nicotine increased TMPRSS2 activity during ALI exposure in the Cultex system. PG/VG decreased activity. JUUL aerosols increased activity (**G**) Changes in activity over 60 min. * Indicate when values became significantly different than the control (*p* ranged from 0.05 to 0.0001). (**H**) Activity at the 60-min timepoint. All graphs are plotted as means ± standard deviation of three independent experiments. Following a Box-Cox transformation, **B, D, F** and **H** were analyzed using a one-way ANOVA followed by Tukey’s test to compare means. Black * = significantly different than the control. Colored * and colored lines = significantly different groups. (**C**) and (**G**) were analyzed using a two-way ANOVA followed by Dunnett’s test to compare means to the control. *p < 0.05, **p < 0.01, ***p < 0.001, ****p < 0.0001. Western blots were horizontally cropped from the original blot.

In the cloud chamber, BEAS-2B cells were exposed to 1 puff of aerosol generated from solutions of either PBS or nicotine (0.03 mg/mL or 0.3 mg/mL) in PBS, then allowed to recover for 24 h before Western blotting and analysis of proteolytic activities (Fig. 3A–D). Nicotine exposures did not significantly affect TMPRSS2 levels in Western blots (Fig. 3A, B). However, in the enzymatic activity assay, TMPRSS2 activity was significantly increased by nicotine (Fig. 3C). At the 60-min timepoint of the assay, the activity was significantly different from the control in the 0.3 mg/mL nicotine treated group (Fig. 3D).

In the Cultex system, BEAS-2B cells were exposed to 10 puffs of aerosol containing JUUL “Virginia Tobacco” aerosol, PG/VG only, or PG/VG with nicotine** (**Fig. 3E–H). In Western blots, TMPRSS2 levels did not differ significantly in any of the treatments when compared to the clean air control group (Fig. 3E, F). However, TMPRSS2 activity increased significantly in the two groups containing PG/VG with 6 or 60 mg/mL of nicotine, but did not change significantly in the JUUL aerosol group, which was generated from fluid containing 60 mg/mL of nicotine (Fig. 3G, H). TMPRSS2 activity decreased significantly in the PG/VG group.

These results demonstrate that aerosols containing nicotine plus PG/VG can elevate TMPRSS2 activity in BEAS-2B cells, which may facilitate infection by enabling more rapid cleavage of the viral spike protein after binding to the host cells.

### JUUL fluids and aerosols and nicotine increased viral pseudoparticle infection of BEAS-2B cells in submerged cultures and ALI exposures

Experiments were done to determine how JUUL fluids and aerosols, and their components (nicotine and PG/VG) affect infection of BEAS-2B cells. SARS-CoV-2 viral pseudoparticles were constructed using lentivirus as the infecting agent. The pseudoparticles contain SARS-CoV-2 spike protein in their envelope and a reporter plasmid that encodes ZsGreen, which is used to identify infected cells.

**Figure 4:**
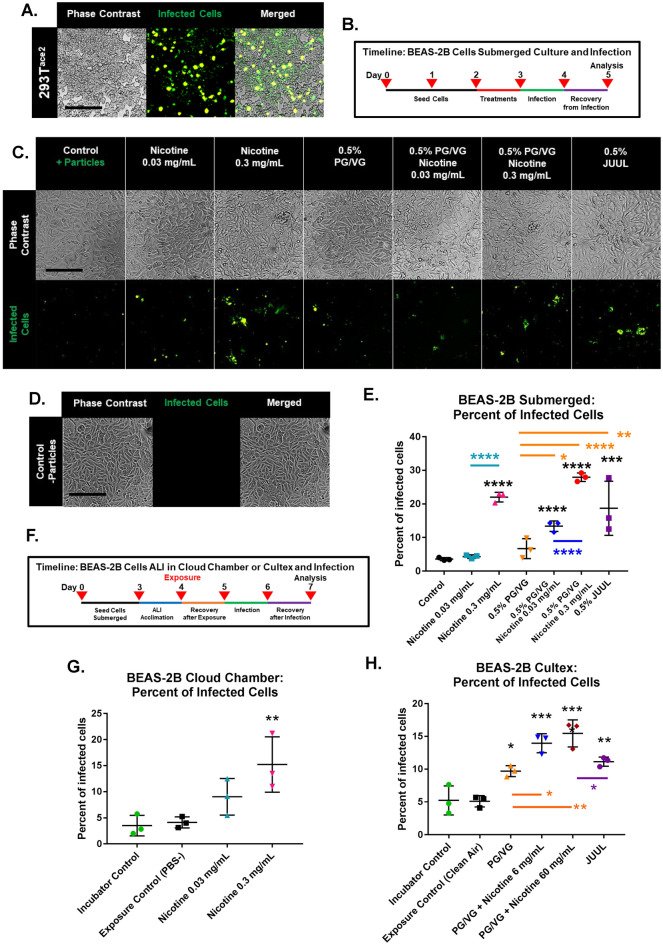
JUUL aerosols and nicotine increased infection after submerged treatment or ALI exposure to aerosols. (**A**) 293T^ACE2^ cells infected by SARS-CoV-2 viral pseudoparticles. Infected cells express ZsGreen. Scale bar = 250 µm. (**B**) Experimental treatments in submerged culture followed by infection. (**C–E**) Micrographs and flow cytometry showing infection of cells treated 24-h with EC chemicals or JUUL fluid then infected for 24 h. (**C, D**) Micrographs showing (**C**) cells infected with pseudoparticles and (**D**) uninfected cells. (**E**) Percent of infected cells determined by flow cytometry. Scale bars in C and D = 250 µm. (**F**) Experimental treatment used with cells exposed at the ALI in a cloud chamber or a Cultex system, then infected with viral pseudoparticles for 24 h. (**G**) Percent of infected cells after cloud chamber exposure to 1 puff of PBS (control), 0.03 mg/mL nicotine, or 0.3 mg/mL nicotine, followed by 24 h of exposure to pseudoparticles. (**H**) Percent of infected cells after exposure to 10 puffs of clean air or EC aerosols, followed by 24 h of exposure to pseudoparticles. Exposure was at the ALI in a Cultex system. All graphs are plotted as the mean ± standard deviation of three independent experiments. Box-Cox transformed data were analyzed using a one-way ANOVA followed by Tukey’s test to compare means. Black * = significantly different than the control in (**E**) or the exposure control in (**G**) and (**H**). Colored * and lines = significantly different groups. *p < 0.05, **p < 0.01, ***p < 0.001, ****p < 0.0001.

293T cells, genetically modified by stable transfection to overexpress ACE2 (293T^ACE2^), were used to test viral pseudoparticle infectivity, determine pseudoparticle density, and optimize the expression of ZsGreen. 293T^ACE2^ cells were infected using an MOI of 0.5 (Fig. 4A). These data were used to optimize infection of BEAS-2B cells (Fig. 4B–H).

After 24 h of submerged treatment, viral pseudoparticles were added to the cell cultures for 24 h, then fluorescence was examined using microscopy and flow cytometry (Fig. 4B). Infection increased significantly in cells treated with JUUL “Virginia Tobacco” fluid (Fig. 4C, E). Nicotine alone (0.3 mg/mL) and both concentrations of nicotine plus PG/VG significantly increased infection (Fig. 4C, E). Infection did not change significantly in cells treated with PG/VG alone.

Similar results were obtained with BEAS-2B cells exposed to nicotine-containing aerosols at the ALI in a cloud chamber (Fig. 4G). Infection was significantly elevated in the group exposed to 0.3 mg/mL of nicotine (Fig. 4G). ALI exposure to various aerosols in the Cultex system significantly increased infections in all treatment groups with the effect being strongest in the groups that had nicotine (Fig. 4H). While infection increased significantly in both the JUUL aerosols and the high nicotine plus PG/VG group, there was a significant difference between these group with the JUUL exposed cells having lower infection. These increases in infection may be due to higher ACE2 expression (Fig. 1) and elevated TMPRSS2 activities (Figs. 2–3) in the nicotine-treated BEAS-2B cells, than in the PG/VG and JUUL groups.

## Discussion

**Figure 5:**
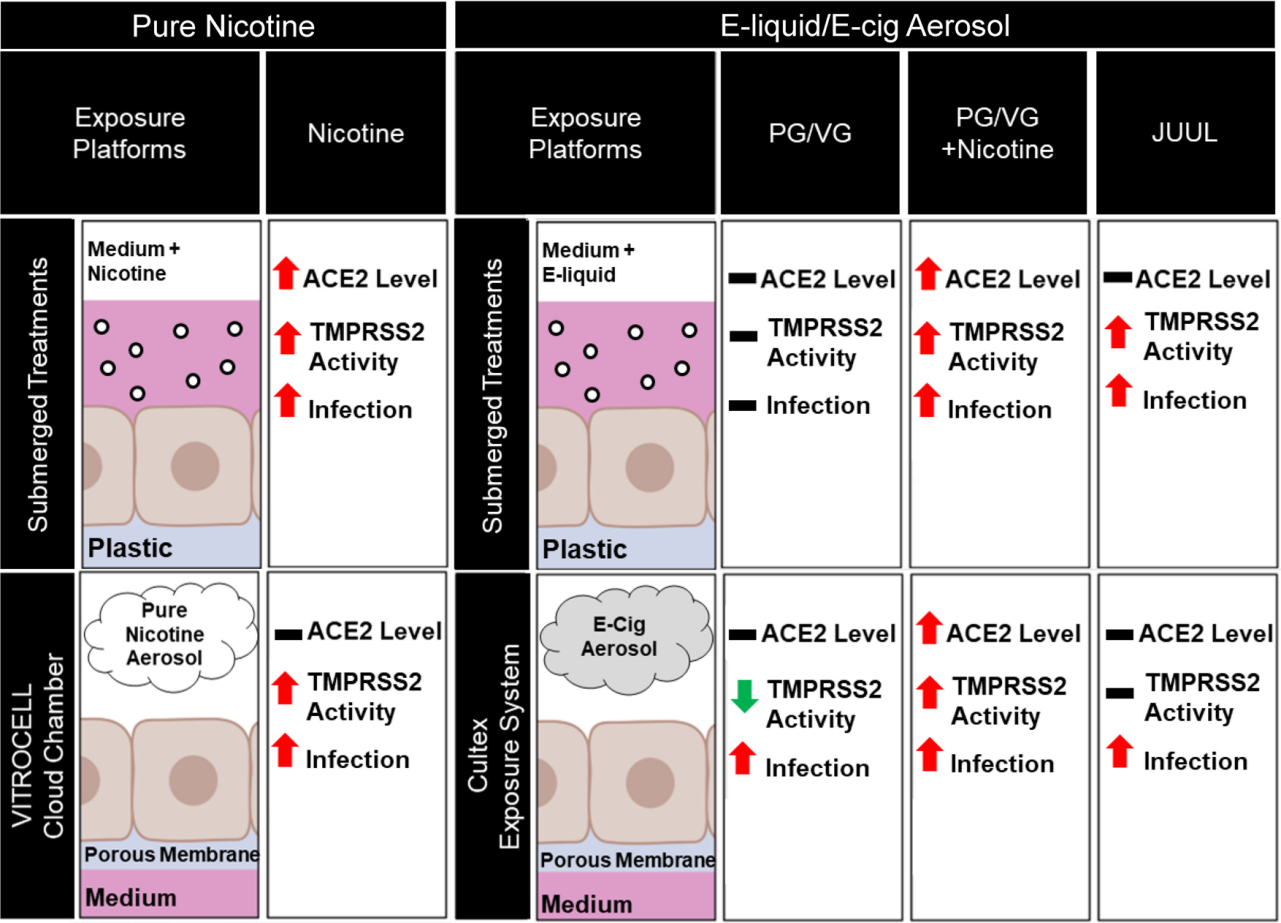
Relationship between JUUL E-liquid, nicotine, and PG/VG exposure and ACE2, TMPRSS2 activity, and viral pseudoparticle infection. Diagram summarizing the major findings in BEAS-2B cells following exposure to JUUL, mixtures (PG/VG plus nicotine), and individual chemicals (nicotine and PG/VG solvent) in three exposure models: submerged treatment (top), air–liquid interface (ALI) exposure in the cloud chamber (bottom left), and ALI exposure to EC aerosols in a Cultex system (bottom right). Increases in ACE2 were only seen in groups containing nicotine or nicotine plus PG/VG. TMPRSS2 activity increased in all groups, except in Cultex aerosols made from PG/VG or JUUL “Tobacco pods”. These two Cultex groups had lower pHs, which likely reduced TMPRSS2 activity. Infection increased in all treatments, except PG/VG in submerged culture. Red arrows indicate a significant increase. Green arrows indicate a significant decrease from the control.

Our goal was to examine the effects of JUUL “Virginia Tobacco” and its individual chemicals on ACE2, TMPRSS2, and infection of BEAS-2B cells by the SARS-CoV-2 virus. JUUL “Virginia Tobacco” was chosen because its concentrations of nicotine and PG/VG are known, and it has relatively few flavor chemicals, which are used at low concentrations ^36^. Our experiments compared JUUL “Virginia Tobacco” aerosols to aerosols made with fluids containing PG/VG, nicotine, and/or PG/VG/nicotine mixtures using chemical concentrations that replicated those in JUUL “Virginia Tobacco” fluid. Our results, summarized in Fig. 5, show the effects of exposure protocol (submerged vs ALI), heated vs unheated aerosols, and JUUL aerosol versus individual aerosol chemicals on ACE2 levels, TMPRSS2 activity, and SARS-CoV-2 pseudoparticle infection. By examining different exposure protocols (submerged vs cloud chamber vs Cultex) and chemical variables (fluids, aerosols, PG/VG, and nicotine), we show that the effects of ECs on viral infection depend on both the context in which exposures occur (submerged vs ALI) and the chemical formulations of the aerosols per se. Three major conclusions can be drawn from these data. First, increases in ACE2 were only seen in groups containing nicotine or nicotine + PG/VG, and not all nicotine containing groups showed this increase (e.g., JUUL). Watermelon-flavored refill fluid with nicotine also increased ACE2 transcripts in BEAS-2B cells ^35^, but the effect was also observed without nicotine, suggesting additional factors in the Watermelon product may increase ACE2.

Secondly, TMPRSS2 activity increased in all groups, except in PG/VG only groups and Cultex aerosols made from JUUL “Tobacco”. These groups had lower pHs that likely reduced TMPRSS2 activity, which has an optimum above 7^52^. Most prior SARS-CoV-2 studies have focused on ACE2 expression^20,30,31,33,35^, and not the effects of EC chemicals on TMPRSS2 levels and activity. Increased TMPRSS2 activity would promote spike cleavage at the S2’ site and fully activate the viral fusion process^12,54^. Our data show that PG/VG, nicotine, and JUUL aerosols had different effects on TMPRSS2 activity. In most cases, TMPRSS2 activities were significantly increased by nicotine. An interesting exception was the lack of effect of JUUL “Virginia Tobacco” aerosols on TMPRSS2 activity, even though JUUL aerosol had a nicotine concentration similar to that in the group with 60 mg/mL of pure nicotine + PG/VG.

Third, infection increased in all treatments, except for PG/VG in submerged culture. In contrast, PG/VG when heated and tested as an aerosol at the ALI with the Cultex platform did increase infection. The increase in infection by aerosolized PG/VG would have been missed had only submerged cultures been used. This “discrepancy” is likely due to the generation of reaction products (e.g., aldehydes, ketones and alcohols) from the solvents during heating^45,55–58^, which in turn enhanced infection in both the PG/VG and JUUL Cultex groups. Metals (e.g., zinc, copper, iron, lead, nickel, tin) are also added to aerosols during heating^47,49,50^, and these could also influence infection. The lack of effect on infection in the PG/VG submerged culture and increased infection with PG/VG in the Cultex system demonstrates the importance of testing heated aerosols at the ALI when working with ECs.

While JUUL aerosols in the Cultex increased infection significantly, this increase was not as great as in the group containing only PG/VG + nicotine in concentrations equivalent to those in the JUUL group (60 mg/mL) (Fig. 4). This result demonstrates that JUUL aerosols significantly decreased the enhancement of infection by nicotine. These data clearly show that the chemicals in the mixture being tested is important and that infection is modulated (in this case decreased) by factors other than nicotine. In the Cultex exposures, both ACE2 and TMPRSS2 increased in the PG/VG + nicotine group, but not in the JUUL group. These data may also explain why infection was significantly higher in the PG/VG/nicotine group than in JUUL (Fig. 4). Other factors in JUUL may have also lowered infection relative to nicotine alone, such as benzoic acid and/or flavor chemicals.

Increases in infection were not always associated with increases in ACE2 and TMPRSS2 (e.g., in the JUUL Cultex group) (Fig. 5). The levels of ACE2 and the activity of TMPRSS2 varied in different treatments. This variation suggests that different mechanisms may be involved in increasing infection. For example, the decrease in TRPRSS2 activity in the PG/VG Cultex group was accompanied by an increase in infection, which may have occurred by a mechanism not involving TMPRSS2, such as endocytosis^34^.

The nicotine concentrations in the fluid of submerged cultures and in insert culture media after ALI exposures were compared to estimated nicotine concentrations in human alveolar lining fluid after smoking a cigarette (Table 1)^59,61^. Exposures were adjusted in the two ALI platforms to produce similar final concentrations of nicotine in the insert fluids after exposure. As seen in Table 1, the nicotine concentrations in both the Cultex and cloud chamber inserts were similar and were within the range reported in the alveolar lining fluid of smokers after smoking one cigarette^59,60^, supporting the conclusion that our ALI exposures were representative of those received by humans using tobacco products. The nicotine concentration (60 mg/mL) found in JUUL ECs was used in the Cultex system and was higher than that used in the other platforms. The Cultex aerosol was diluted before entering the exposure chamber to ensure uniform distribution, therefore the amount of nicotine reaching each insert was less than in the parent EC fluid. In contrast, undiluted aerosol was used in the cloud chamber, where it disperses uniformly enabling a lower starting concentration and puff number to be used. These data demonstrate that our chemical exposures were similar between the cloud chamber and Cultex system and that exposures in the ALI systems, based on nicotine concentration, were within the range a tobacco product user would have in their alveolar lining fluid.

**Table 1:** Nicotine concentrations across the in vitro platforms compared to nicotine concentrations in alveolar lining fluid of human smokers^1^.

Platform	Concentration in submerged culture fluid and aerosolized fluid	Concentration in culture fluid and insert fluid	Concentration in alveolar lining fluid of smokers after 1 cigarette^1^
Submerged culture	30 and 300 µg/mL	30 and 300 µg/mL	1–10 µg/mL
Cloud chamber ALI exposure	30 and 300 µg/mL	1.3 µg/mL	1–10 µg/mL
Cultex ALI exposure	6 and 60 mg/mL	1.5 µg/mL Authentic JUUL 1.8 µg/mL Lab-made Nicotine	1–10 µg/mL

^1^Ranges are taken from references in the^57,58^.

The effective concentrations of nicotine and the length of exposure to nicotine differed across platforms. Nicotine increased SARS-CoV-2 pseudoparticle infection of BEAS-2B cells in all exposure systems. In submerged culture, nicotine concentrations were higher and exposures were continuous over 24 h, in contrast to the ALI systems, in which the lower nicotine concentrations were intermittent and short in duration, suggesting cells were more sensitive when exposed at the ALI. Our data agree with other studies showing that ALI exposure of A549 cells to zinc nanoparticles was more likely to produce an effect than exposure in submerged cultures^62^ and that gaseous exposure to aldehydes at the ALI caused significantly higher levels of IL-8 secretion than exposures in submerged culture^63^. The apparent increase in cell sensitivity at the ALI may come about because nicotine or other chemicals in the EC aerosols were not diluted by the medium and interact directly with the surface of cells in the absence of culture medium. Because infection varied with nicotine concentrations, results of ALI and submerged exposures are likely to vary with EC products, which are available in a broad range of nicotine concentrations^25,64^.

Our in vitro models support the conclusion that cells are more responsive to nicotine at the ALI than when submerged and that nicotine alone increased ACE2 levels (except in the cloud chamber), TMPRSS2 activity, and SARS-CoV-2 pseudoparticle infection. ALI exposure with the Cultex system is preferable to submerged culture since it more closely resembles actual human exposure during vaping, apparently is more sensitive, and can detect changes that occur during heating (PG/VG submerged vs PG/VG Cultex).

In summary, our data show that nicotine, nicotine + PG/VG, and JUUL “Virginia Tobacco” aerosols increased SARS-CoV-2 infection in BEAS-2B cells. In the Cultex, nicotine + PG/VG alone was more effective than JUUL aerosol, showing that chemicals in JUUL aerosols can reduce nicotine’s effect. In ALI exposures, increases in infectivity were not necessarily correlated with increases in ACE2 concentrations and TMPRRS2 activity, suggesting that more than one mechanism increased SARS-CoV-2 infection in the different platforms. Infection results were generally similar across the three exposure platforms, but the cells were more sensitive to exposures in the Cultex system. Given the highly variable nature of EC devices and their fluids^66^, it is probable that infection by SARS-CoV-2 is influenced by the product, vaper’s topography, and the e-liquid used for aerosol generation. This variability has likely contributed to differing conclusions in prior studies on tobacco products and their relationship to COVID-19 ^5–7,13–18,67^. EC products have nicotine concentrations ranging from 0 to 60 mg/mL ^21,22,63,68^, operate at different powers, have different atomizer designs^69,70^ and have varying chemical mixtures in their fluids, which can all affect SARS-CoV-2 infection and severity. Based on our BEAS-2B cell data, it is probable that some EC products deliver sufficient nicotine to increase infection, while those that are nicotine-free or have low concentrations of nicotine may not affect SARS-CoV-2 infection. Our data show that numerous variables affect the relationship between EC use and COVID-19. Understanding these variables can be valuable in improving EC safety. For example, capping the allowable concentration of nicotine in e-liquids may reduce the likelihood of SARS-CoV-2 infection in EC users.

### Limitations of the study

We examined the effect of EC aerosols on BEAS-2B cells. In the future, it will be important to extend these studies to a 3D organotypic model of the respiratory epithelium that closely resembles *in-vivo* exposure in humans. Our study is limited to one SARS-CoV-2 variant and could in the future be extended to new variants as they arise. Our work included JUUL “Virginia Tobacco”, which operates with a relatively low voltage and power. Variations in power can affect formation of reaction products, which may in turn alter infectability of cells. Finally, our exposures were acute and could be extended to chronic exposure in the future.

## Supplementary Information

Supplementary Information.

## Data Availability

The datasets analyzed in this are available from the corresponding author upon request.

## Abbreviations

EC: Electronic cigarettes
PG: Propylene glycol
VG: Vegetable glycerin
ALI: Air–liquid- interface
ACE2: Angiotensin converting enzyme 2
TMPRSS2: Transmembrane serine protease 2
COVID-19: Corona virus disease-2019
SARS-CoV-2: Severe acute respiratory syndrome coronavirus 2
MOI: Multiplicity of infection

## References

[1] World Health Organization (WHO). Data stories. Global excess deaths associated with covid-19, January 2020—December 2021. https://www.who.int/data/stories/global-excess-deaths-associated-with-covid-19-january-2020-december-2021. Accessed 10 July (2022).

[2] World Health Organization (WHO). WHO coronavirus (COVID-19) dashboard. https://covid19.who.int/. Accessed 10 July (2022).

[3] Arcavi L, Benowitz NL (2004). Cigarette smoking and infection. Arch. Intern. Med..

[4] U.S. Department of Health and Human Services, Center for Disease Control and Prevention, National Center for Chronic Disease Prevention and Health Promotion, Office on Smoking and Health. Let’s make the next generation tobacco-free: Your guide to the 50th anniversary surgeon general’s report on smoking and health. Rockville (MD): US Department of Health and Human Services, Public Health Service, Office of the Surgeon General (US); (2014).

[5] Simons D, Shahab L, Brown J, Perski O (2021). The association of smoking status with SARS-CoV-2 infection, hospitalization and mortality from COVID-19: A living rapid evidence review with Bayesian meta-analyses (version 7). Addiction.

[6] Changeux JP, Amoura Z, Rey FA, Miyara M (2020). A nicotinic hypothesis for Covid-19 with preventive and therapeutic implications. C R Biol..

[7] Farsalinos K, Bagos PG, Giannouchos T, Niaura R, Barbouni A, Poulas K (2021). Smoking prevalence among hospitalized COVID-19 patients and its association with disease severity and mortality: An expanded re-analysis of a recent publication. Harm. Reduct. J..

[8] Patanavanich R, Glantz SA (2020). Smoking is associated with COVID-19 progression: A meta-analysis. Nicotine Tob. Res..

[9] Jackson SE, Brown J, Shahab L, Steptoe A, Fancourt D (2021). COVID-19, smoking and inequalities: A study of 53 002 adults in the UK. Tob. Control.

[10] Hopkinson NS, Rossi N, El-Sayed MJ, Laverty AA, Quint JK, Freidin M, Visconti A, Murray B, Modat M, Ourselin S, Small K, Davies R, Wolf J, Spector TD, Steves CJ, Falchi M (2021). Current smoking and COVID-19 risk: Results from a population symptom app in over 2.4 million people. Thorax.

[11] Shastri MD, Shukla SD, Chong WC, Kc R, Dua K, Patel RP, Peterson GM, O’Toole RF (2021). Smoking and COVID-19: What we know so far. Respir. Med..

[12] Hoffmann M, Kleine-Weber H, Schroeder S, Krüger NHT, Erichsen S, Schiergens TS, Herrler G, Wu NH, Nitsche A, Müller MA, Drosten C, Pöhlmann S (2020). (2020) SARS-CoV-2 cell entry depends on ACE2 and TMPRSS2 and is blocked by a clinically proven protease inhibitor. Cell.

[13] Smith JC, Sausville EL, Girish V, Yuan ML, Vasudevan A, John KM, Sheltzer JM (2020). Cigarette smoke exposure and inflammatory signaling increase the expression of the SARS-CoV-2 receptor ACE2 in the respiratory tract. Dev. Cell.

[14] Zhang J-Y, Wang X-M, Xing X, Xu Z, Zhang C, Song J-W, Fan X, Xia P, Fu J-L, Wang S-Y, Xu R-N, Dai X-P, Shi L, Huang L, Jiang T-J, Shi M, Zhang Y, Zumla A, Maeurer M (2020). Single-cell landscape of immunological responses in patients with COVID-19. Nat. Immunol..

[15] Leung JM, Yang CX, Tam A, Shaipanich T, Hackett T-L, Singhera GK, Dorscheid DR, Sin DD (2020). ACE-2 expression in the small airway epithelia of smokers and COPD patients: Implications for COVID-19. Eur. Respir. J..

[16] Cai G, Bossé Y, Xiao F, Kheradmand F, Amos CI (2020). Tobacco smoking increases the lung gene expression of ACE2, the receptor of sars-cov-2. Am. J. Respir. Crit. Care Med..

[17] Brake SJ, Barnsley K, Lu W, McAlinden KD, Eapen MS, Sohal SS (2020). Smoking upregulates angiotensin-converting enzyme-2 receptor: A potential adhesion site for novel coronavirus SARS-CoV-2 (Covid-19). J. Clin. Med..

[18] Muus C, Luecken MD, Eraslan G, Sikkema L, Waghray A, Heimberg G, Kobayashi Y, Vaishnav ED, Subramanian A, Smillie C, Jagadeesh KA, Duong ET, Fiskin E, Torlai Triglia E, Ansari M, Cai P, Lin B, Buchanan J, Chen S (2021). Single-cell meta-analysis of SARS-CoV-2 entry genes across tissues and demographics. Nat. Med..

[19] Purkayastha A, Sen C, Garcia G, Langerman J, Shia DW, Meneses LK, Vijayaraj P, Durra A, Koloff CR, Freund DR, Chi J, Rickabaugh TM, Mulay A, Konda B, Sim MS, Stripp BR, Plath K, Arumugaswami V, Gomperts BN (2020). Direct exposure to sars-cov-2 and cigarette smoke increases infection severity and alters the stem cell-derived airway repair response. Cell Stem Cell.

[20] Ghosh A, Girish V, Yuan ML, Coakley RD, Wrennall JA, Alexis NE, Sausville EL, Vasudevan A, Chait AR, Sheltzer JM, Tarran R (2022). Combustible and electronic cigarette exposures increase ACE2 activity and SARS-CoV-2 spike Binding. Am. J. Respir. Crit. Care Med..

[21] Trtchounian A, Talbot P (2011). Electronic nicotine delivery systems: Is there a need for regulation?. Tob. Control.

[22] National Academies of Sciences E, and Medicine; Health and Medicine Division; Board on Population Health and Public Health Practice; Committee on the Review of the Health Effects of Electronic Nicotine Delivery Systems. Public Health Consequences of E-Cigarettes. In: KL EDL, Stratton K, editors. Toxicology of E-Cigarette Constituents. Volume 5, edn. Washington (DC): National Academies Press (US); 2018. 29894118

[23] Becker TD, Arnold MK, RoV ML, Rice TR (2021). Systematic review of electronic cigarette use (Vaping) and mental health comorbidity among adolescents and young adults. Nicot. Tob. Res..

[24] Hua M, Omaiye EE, Luo W, McWhirter KJ, Pankow JF, Talbot P (2019). Identification of cytotoxic flavor chemicals in top-selling electronic cigarette refill fluids. Sci. Rep..

[25] Omaiye EE, Luo W, McWhirter KJ, Pankow JF, Talbot P (2020). Electronic cigarette refill fluids sold worldwide: Flavor chemical composition, toxicity, and hazard analysis. Chem. Res. Toxicol..

[26] Pisinger C, Døssing M (2014). A systematic review of health effects of electronic cigarettes. Prev. Med..

[27] Gotts JE, Jordt S-E, McConnell R, Tarran R (2019). What are the respiratory effects of e-cigarettes?. BMJ.

[28] Hua M, Talbot P (2016). Potential health effects of electronic cigarettes: A systematic review of case reports. Prev. Med. Rep..

[29] Hua M, Sadah S, Hristidis V, Talbot P (2020). Health effects associated with electronic cigarette use: Automated mining of online forums. J. Med. Internet Res..

[30] Masso-Silva JA, Moshensky A, Shin J, Olay J, Nilaad S, Advani I, Bojanowski CM, Crotty S, Li WT, Ongkeko WM, Singla S, Crotty Alexander LE (2021). Chronic E-cigarette aerosol inhalation alters the immune state of the lungs and increases ACE2 expression, raising concern for altered response and susceptibility to SARS-CoV-2. Front. Physiol..

[31] Wang Q, Sundar IK, Li D, Lucas JH, Muthumalage T, McDonough SR, Rahman I (2020). E-cigarette-induced pulmonary inflammation and dysregulated repair are mediated by nAChR α7 receptor: Role of nAChR α7 in SARS-CoV-2 Covid-19 ACE2 receptor regulation. Respir. Res..

[32] Lallai V, Manca L, Fowler CD (2021). E-cigarette vape and lung ACE2 expression: Implications for coronavirus vulnerability. Environ. Toxicol. Pharmacol..

[33] Naidu V, Zeki AA, Sharma P (2021). Sex differences in the induction of angiotensin converting enzyme 2 (ACE-2) in mouse lungs after e-cigarette vapor exposure and its relevance to COVID-19. J. Investig. Med..

[34] Jackson CB, Farzan M, Chen B, Choe H (2022). Mechanisms of SARS-CoV-2 entry into cells. Nat. Rev. Mol. Cell Biol..

[35] McAlinden KD, Lu W, Ferdowsi PV, Myers S, Markos J, Larby J, Chia C, Weber HC, Haug G, Eapen MS, Sohal SS (2021). Electronic cigarette aerosol is cytotoxic and increases ACE2 expression on human airway epithelial cells: Implications for SARS-CoV-2 (COVID-19). J. Clin. Med..

[36] Omaiye EE, McWhirter KJ, Luo W, Tierney PA, Pankow JF, Talbot P (2019). High concentrations of flavor chemicals are present in electronic cigarette refill fluids. Sci. Rep..

[37] US Food and Drug Administration (FDA). Press announcement. FDA finalizes enforcement policy on unauthorized flavored cartridge-based e-cigarettes that appeal to children, including fruit and mint. https://www.fda.gov/news-events/press-announcements/fda-finalizes-enforcement-policy-unauthorized-flavored-cartridge-based-e-cigarettes-appeal-children. Accessed on 10 July 2022.

[38] Bahl V, Lin S, Xu N, Davis B, Wang Y, Talbot P (2012). Comparison of electronic cigarette refill fluid cytotoxicity using embryonic and adult models. Reprod. Toxicol..

[39] Behar RZ, Bahl V, Wang Y, Lin S, Xu N, Davis B, Talbot P (2012). A method for rapid dose–response screening of environmental chemicals using human embryonic stem cells. J. Pharmacol. Toxicol. Methods.

[40] Behar RZ, Luo W, McWhirter KJ, Pankow JF, Talbot P (2018). Analytical and toxicological evaluation of flavor chemicals in electronic cigarette refill fluids. Sci. Rep..

[41] Omaiye EE, Luo W, McWhirter KJ, Pankow JF, Talbot P (2022). Disposable puff bar electronic cigarettes: Chemical composition and toxicity of e-liquids and a synthetic coolant. Chem. Res. Toxicol..

[42] Smart DJ, Phillips G (2021). Collecting e-cigarette aerosols for in vitro applications: A survey of the biomedical literature and opportunities to increase the value of submerged cell culture-based assessments. J. Appl. Toxicol..

[43] Nair V, Tran M, Behar RZ, Zhai S, Cui X, Phandthong R, Wang Y, Pan S, Luo W, Pankow JF, Volz DC, Talbot P (2020). Menthol in electronic cigarettes: A contributor to respiratory disease?. Toxicol. Appl. Pharmacol..

[44] Kosmider L, Sobczak A, Fik M, Knysak J, Zaciera M, Kurek J, Goniewicz ML (2014). Carbonyl compounds in electronic cigarette vapors: Effects of nicotine solvent and battery output voltage. Nicotine Tob. Res..

[45] Uchiyama S, Noguchi M, Sato A, Ishitsuka M, Inaba Y, Kunugita N (2020). Determination of thermal decomposition products generated from e-cigarettes. Chem. Res. Toxicol..

[46] Talih S, Salman R, El-Hage R, Karam E, Karaoghlanian N, El-Hellani A, Saliba N, Shihadeh A (2019). Characteristics and toxicant emissions of JUUL electronic cigarettes. Tob. Control..

[47] Olmedo P, Goessler W, Tanda S, Grau-Perez M, Jarmul S, Aherrera A, Chen R, Hilpert M, Cohen JE, Navas-Acien A, Rule AM (2018). Metal concentrations in e-cigarette liquid and aerosol samples: The contribution of metallic coils. Environ. Health Perspect..

[48] Williams M, Villarreal A, Bozhilov K, Lin S, Talbot P (2013). Metal and silicate particles including nanoparticles are present in electronic cigarette cartomizer fluid and aerosol. PLoS ONE.

[49] Williams M, Bozhilov K, Ghai S, Talbot P (2017). Elements including metals in the atomizer and aerosol of disposable electronic cigarettes and electronic hookahs. PLoS ONE.

[50] Williams M, Li J, Talbot P (2019). Effects of model, method of collection, and topography on chemical elements and metals in the aerosol of tank-style electronic cigarettes. Sci. Rep..

[51] Brown JE, Luo W, Isabelle LM, Pankow JF (2014). Candy flavorings in tobacco. N. Engl. J. Med..

[52] Shrimp JH, Kales SC, Sanderson PE, Simeonov A, Shen M, Hall MD (2020). An enzymatic TMPRSS2 assay for assessment of clinical candidates and discovery of inhibitors as potential treatment of COVID-19. ACS Pharmacol. Transl. Sci..

[53] Crawford KHD, Eguia R, Dingens AS, Loes AN, Malone KD, Wolf CR, Chu HY, Tortorici MA, Veesler D, Murphy M, Pettie D, King NP, Balazs AB, Bloom JD (2020). Protocol and reagents for pseudotyping lentiviral particles with SARS-CoV-2 spike protein for neutralization assays. Viruses.

[54] Shang J, Wan Y, Luo C, Ye G, Geng Q, Auerbach A, Li F (2020). Cell entry mechanisms of SARS-CoV-2. Proc. Natl. Acad. Sci..

[55] Bitzer ZT, Goel R, Reilly SM, Elias RJ, Silakov A, Foulds J, Muscat J, Richie JP (2018). Effect of flavoring chemicals on free radical formation in electronic cigarette aerosols. Free Radical Biol. Med..

[56] Bitzer ZT, Goel R, Reilly SM, Foulds J, Muscat J, Elias RJ (2018). Richie JP Effects of solvent and temperature on free radical formation in electronic cigarette aerosols. Chem. Res. Toxicol..

[57] Erythropel HC, Davis LM, de Winter TM, Jordt SE, Anastas PT, O’Malley SS, Krishnan-Sarin S, Zimmerman JB (2019). Flavorant–solvent reaction products and menthol in juul e-cigarettes and aerosol. Am. J. Prev. Med..

[58] Jensen RP, Strongin RM, Peyton DH (2017). Solvent chemistry in the electronic cigarette reaction vessel. Sci. Rep..

[59] Eiserich P, Cross E (1995). Dietary antioxidants and cigarette smoke-induced biomolecular damage: A complex interaction. Am. J. Clin. Nutr..

[60] Clunes LA, Bridges A, Alexis N, Tarran R (2008). In vivo versus in vitro airway surface liquid nicotine levels following cigarette smoke exposure. J. Anal. Toxicol..

[61] Hoshino Y, Mio T, Nagai S, Miki H, Ito I, Izumi T (2001). Cytotoxic effects of cigarette smoke extract on an alveolar type II cell-derived cell line. Am. J. Phys-Lung Cell. Mol. Physiol..

[62] Lenz AG, Karg E, Brendel E, Hinze-Heyn H, Maier KL, Eickelberg O, Stoeger T, Schmid O (2013). Inflammatory and oxidative stress responses of an alveolar epithelial cell line to airborne zinc oxide nanoparticles at the air-liquid interface: A comparison with conventional, submerged cell-culture conditions. Biomed. Res. Int..

[63] Dwivedi AM, Upadhyay S, Johanson G, Ernstgård L, Palmberg L (2018). Inflammatory effects of acrolein, crotonaldehyde and hexanal vapors on human primary bronchial epithelial cells cultured at air-liquid interface. Toxicol. In Vitro.

[64] Davis B, Dang M, Kim J, Talbot P (2015). Nicotine concentrations in electronic cigarette refill and do-it-yourself fluids. Nicotine Tob Res..

[65] Maggi F, Rosellini A, Spezia PG, Focosi D, Macera L, Lai M, Pistello M, de Iure A, Tomino C, Bonassi S, Russo P (2021). Nicotine upregulates ACE2 expression and increases competence for SARS-CoV-2 in human pneumocytes. ERJ Open Res..

[66] Cheng T (2014). Chemical evaluation of electronic cigarettes. Tob. Control.

[67] Vardavas C, Nikitara K (2020). COVID-19 and smoking: A systematic review of the evidence. Tob. Induc. Dis..

[68] Omaiye EE, Luo W, McWhirter KJ, Pankow JF, Talbot P (2022). Flavour chemicals, synthetic coolants and pulegone in popular mint-flavoured and menthol-flavoured e-cigarettes. Tob. Control.

[69] Williams M, Talbot P (2019). Design features in multiple generations of electronic cigarette atomizers. Int. J. Environ. Res. Public Health.

[70] Omaiye EE, Williams M, Bozhilov KN, Talbot P (2021). Design features and elemental/metal analysis of the atomizers in pod-style electronic cigarettes. PLoS ONE.

