# Supplementary material for: New insights into how popular electronic cigarette aerosols and aerosol constituents affect SARS-CoV-2 infection of human bronchial epithelial cells
Source: Sci Rep. 2023 Apr 10;13:5807. doi: 10.1038/s41598-023-31592-x (PMC10086046; doi:10.1038/s41598-023-31592-x)

The following slides are the original unedited Western blot strip images used in this paper. The full-length Western blot membranes are not available because our protocol includes a step in which the membrane is cut horizontally into strips. This step: (1) reduces and conserves the total volume of antibody used in each experiment; (2) produces bands with more robust signals than those in whole blots treated with antibody; (3) allows optimization of exposure times for each antibody; and (4) eliminates the need to strip and reprobe blots, which is time consuming and does not yield quantitative data.

We used molecular weight ladders and the expected molecular weight of each protein of interest to determine where to cut the membrane into strips. For each Western blot experiment, two protein standard ladders were loaded in the first and the last well of the gel, to facilitate cutting blot at the appropriate molecular weight. For this manuscript, the membranes were cut at 75kD and slightly below 50kD. The top strip had proteins with molecular weights ranging from 75-250 kD and was labeled with an antibody to ACE2 (molecular weight ~100kD). The second strip had proteins with molecular weights between 50-75 kD and was labeled with an antibody to TMPRSS2 (~70 kD). The last segment had proteins with molecular weights below 50 kD and was labeled with an antibody to GAPDH (molecular weight = 37 kD).

Submerged culture Western blot 1

Membrane 1

Samples order (left to right)

1. Control
2. Nicotine 0.03 mg/mL
3. Nicotine 0.3 mg/mL
4. 0.5% PG/VG
5. 0.5% PG/VG Nicotine 0.03 mg/mL
6. 0.5% PG/VG Nicotine 0.3 mg/mL
7. 0.5% JUUL

Membrane was cut horizontally at 75 kD molecular weight and slightly below 50 kD

**Blot image**	**Blot merged with ladder**

ACE2 (~100 kD)

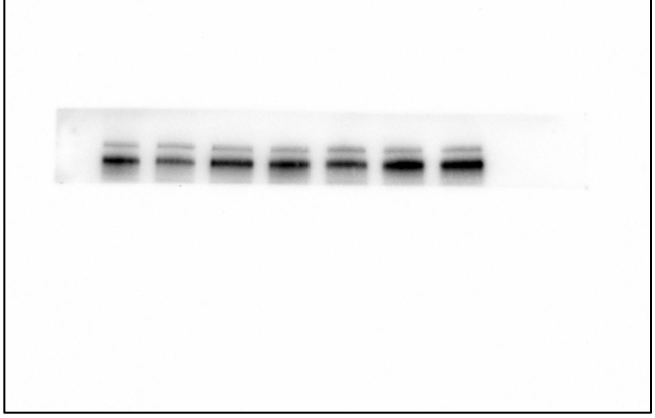

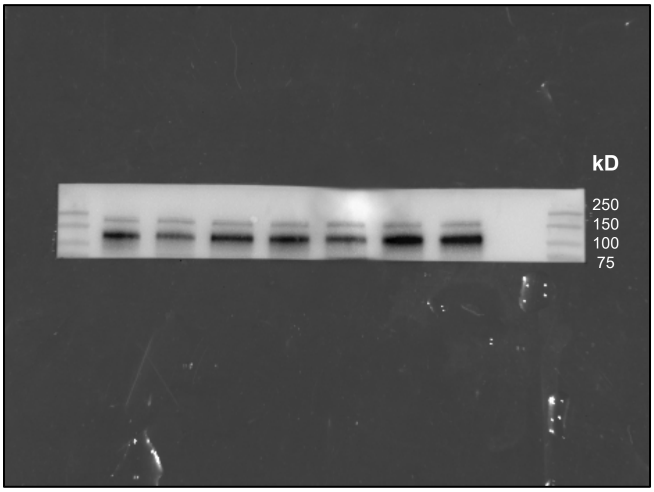

TMPRSS2 (~70 kD)

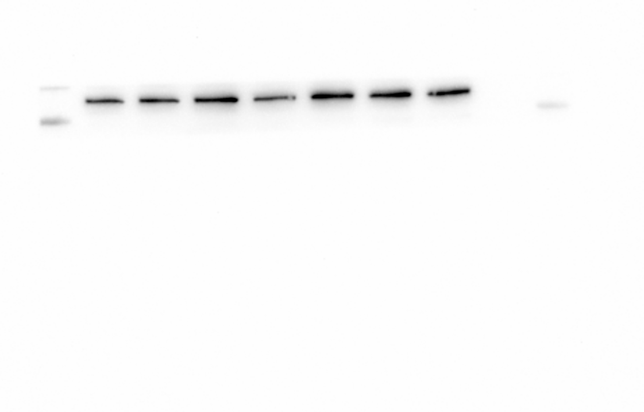

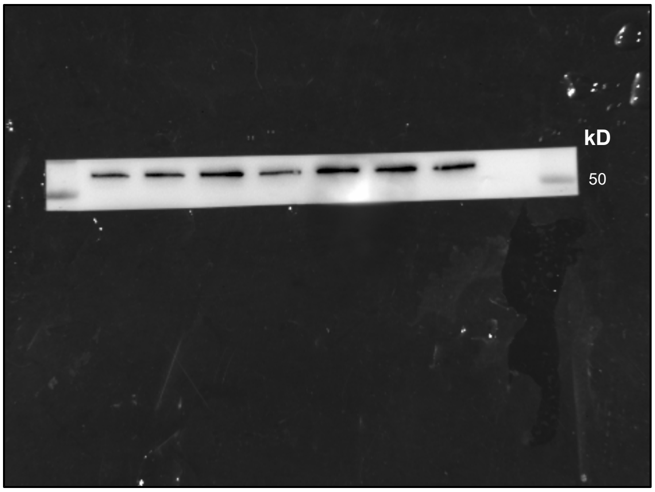

GAPDH (37 kD)

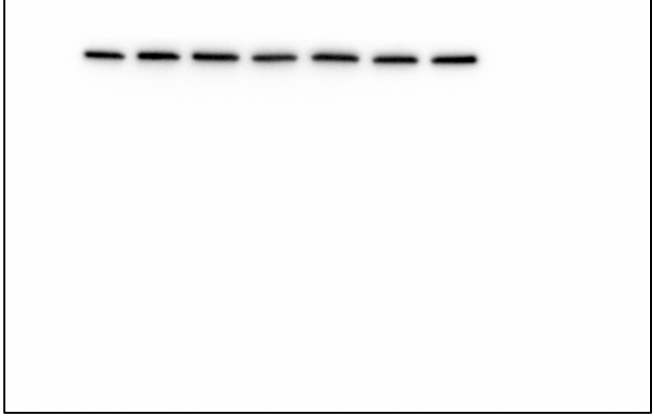

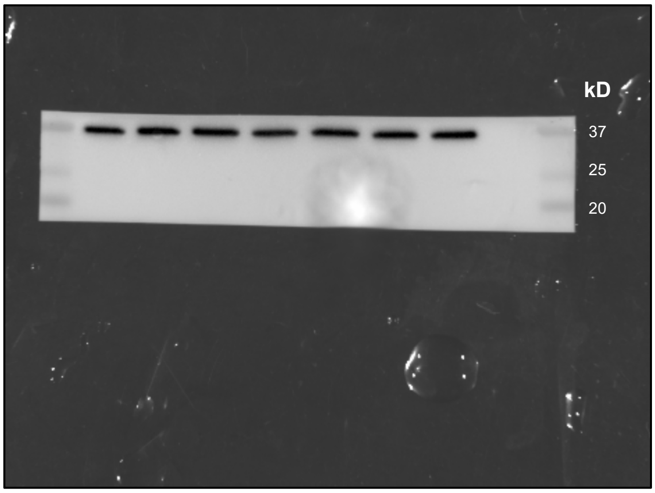

Submerged culture Western blot 2

Membrane 2

Samples order (left to right)
1. Control
2. Nicotine 0.03 mg/mL
3. Nicotine 0.3 mg/mL
4. 0.5% PG/VG
5. 0.5% PG/VG Nicotine 0.03 mg/mL
6. 0.5% PG/VG Nicotine 0.3 mg/mL
7. 0.5% JUUL

Membrane was cut horizontally at 75 kD molecular weight and slightly below 50 kD.

Blot image | Blot merged with ladder

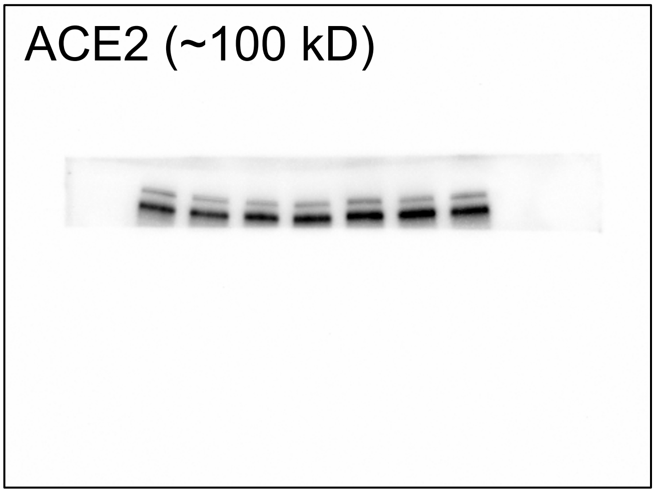

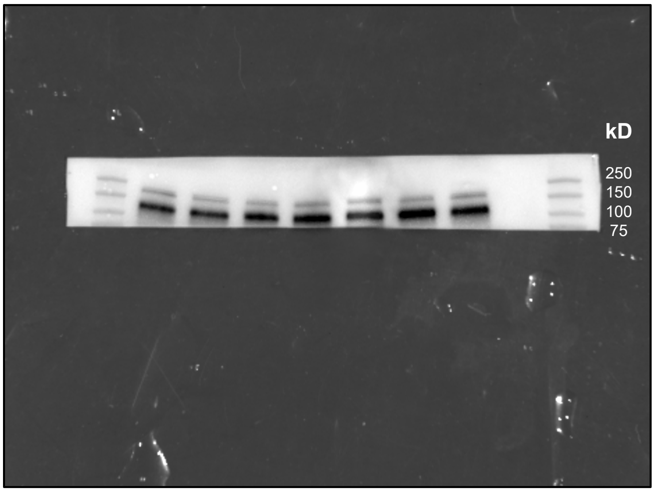

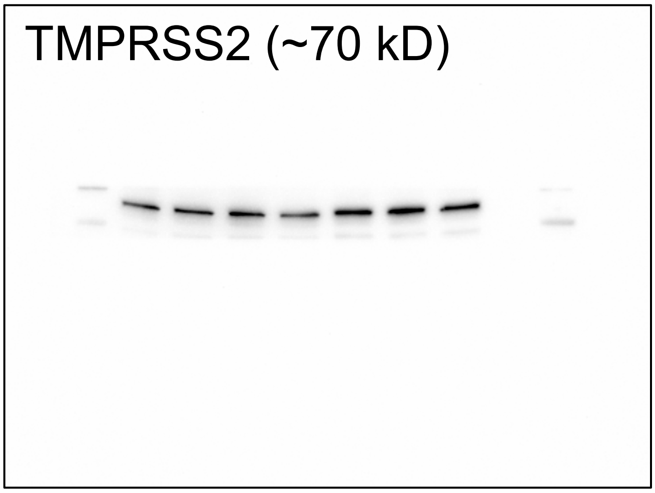

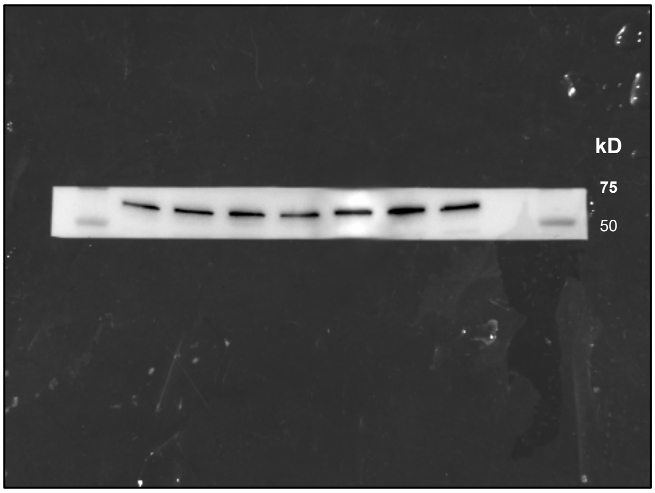

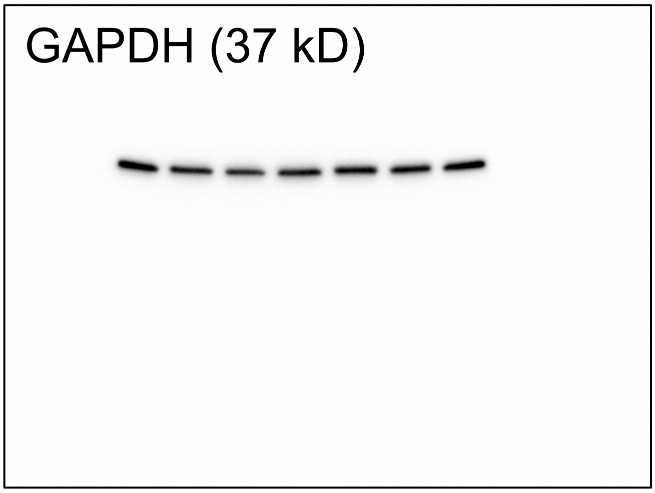

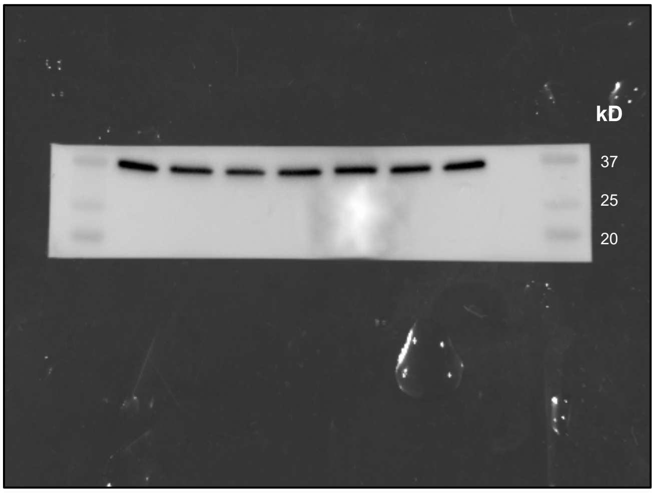

Submerged culture Western blot 3

Membrane 3

Samples order (left to right)

1. Control
2. Nicotine 0.03 mg/mL
3. Nicotine 0.3 mg/mL
4. 0.5% PG/VG
5. 0.5% PG/VG Nicotine 0.03 mg/mL
6. 0.5% PG/VG Nicotine 0.3 mg/mL
7. 0.5% JUUL

Membrane was cut horizontally at 75 kD molecular weight and slightly below 50 kD.

Blot image	Blot merged with ladder

ACE2 (~100 kD)

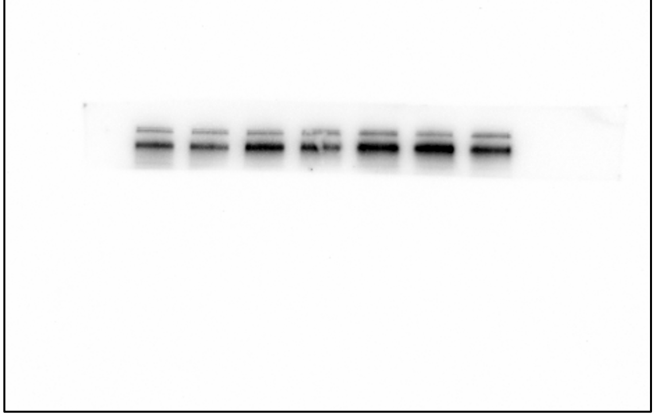

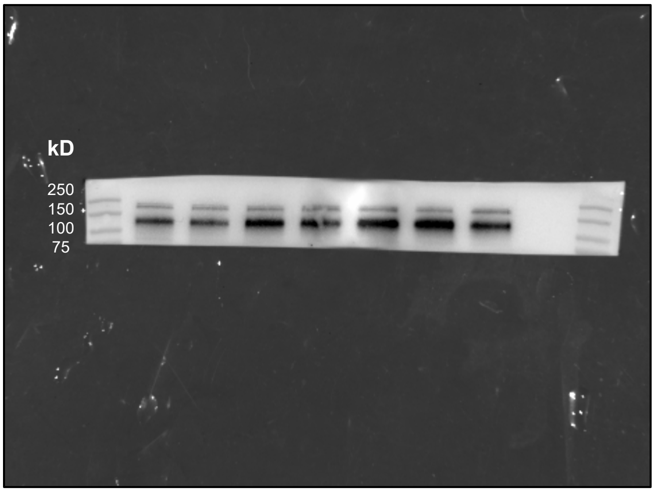

TMPRSS2 (~70 kD)

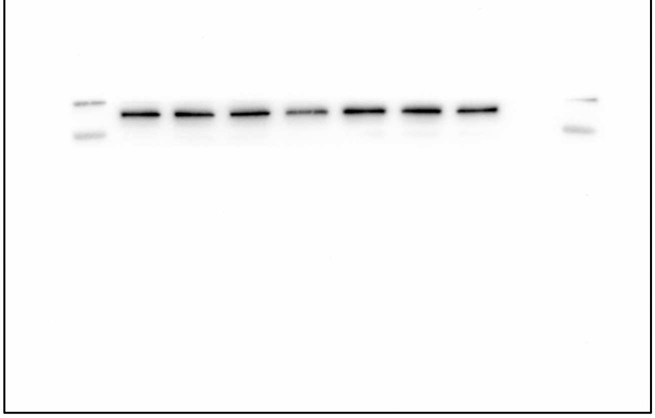

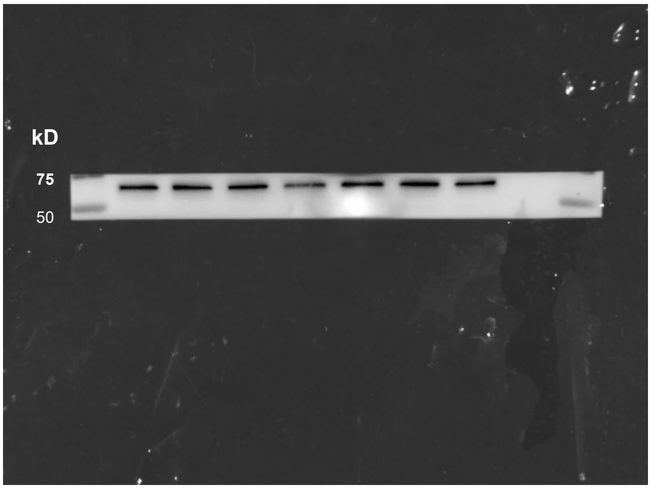

GAPDH (37 kD)

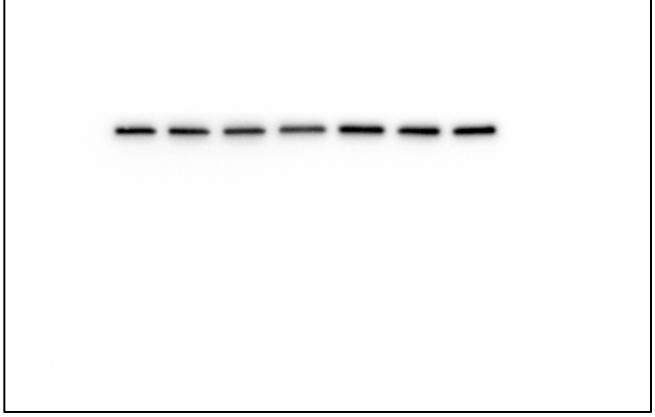

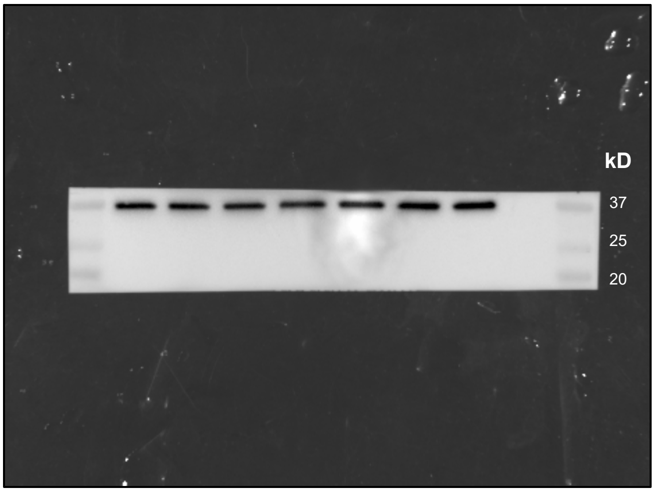

Cloud chamber ALI Western blot 1 and 2

Membrane 1

Samples order (left to right)

Western blot 1:
1. Incubate control
2. PBS- (Exposure control)
3. Nicotine 0.03 mg/mL
4. Nicotine 0.3 mg/mL

Western blot 2:

5. Nicotine 0.3 mg/mL
6. Nicotine 0.03 mg/mL
7. PBS- (Exposure control)
8. Incubate control

Membrane was cut horizontally at 75 kD molecular weight and slightly below 50 kD.

**Blot image**	**Blot merged with ladder**

ACE2 (~100 kD)

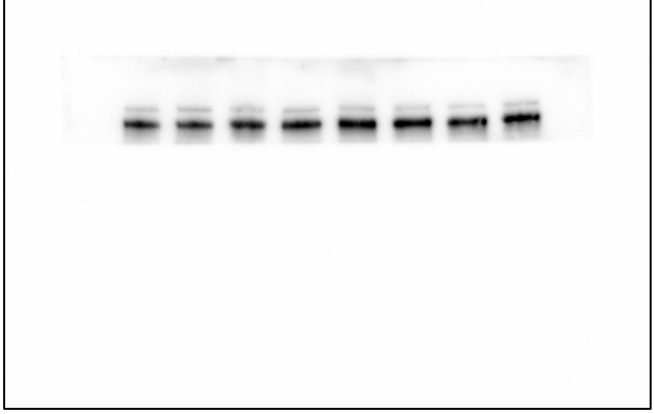

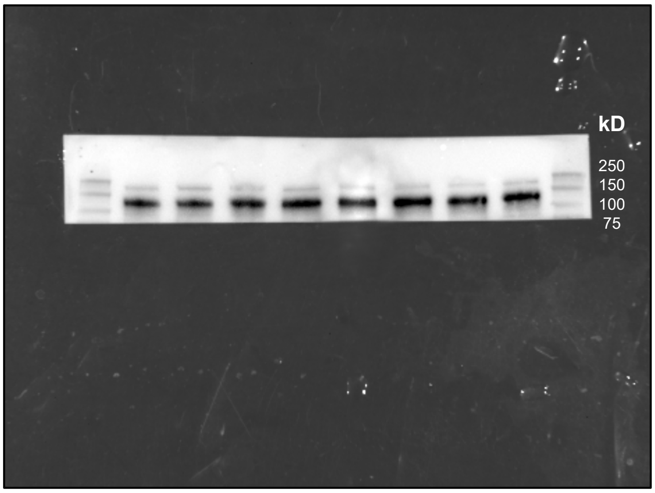

TMPRSS2 (~70 kD)

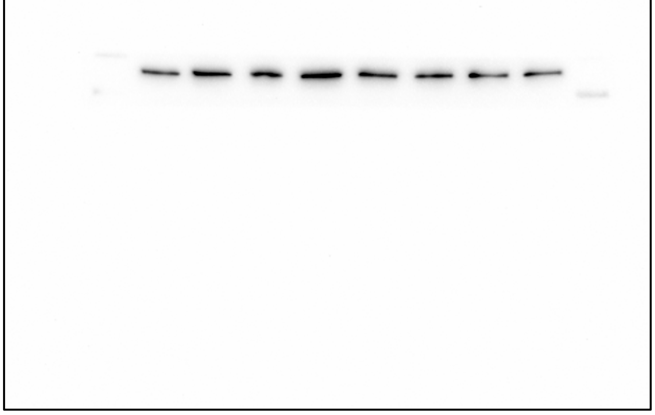

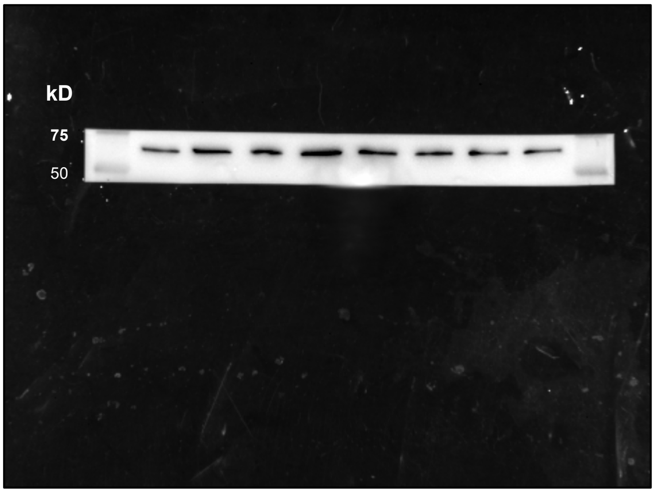

GAPDH (37 kD)

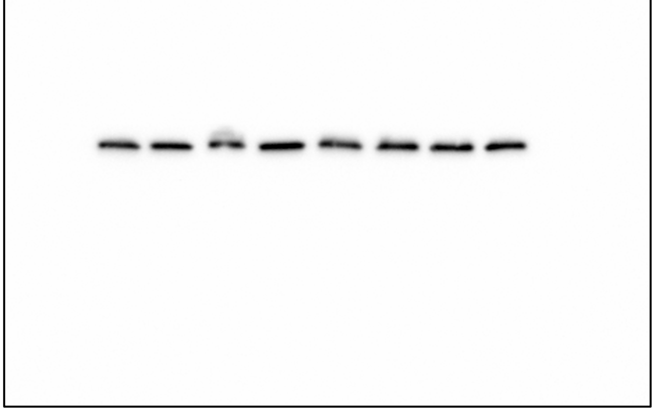

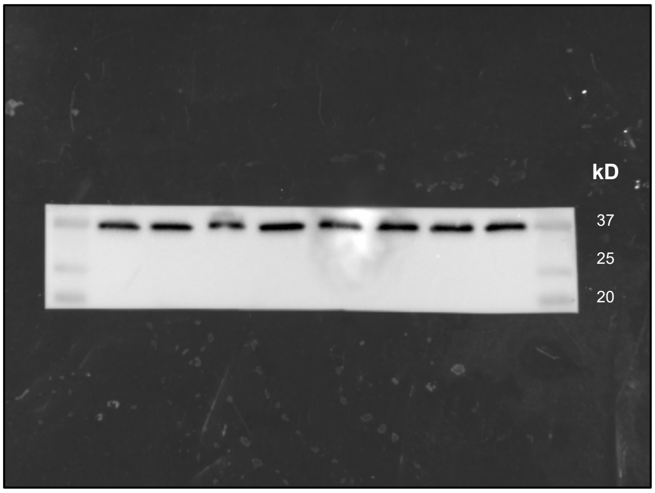

Cloud chamber ALI Western blot 3

Membrane 2

Samples order (left to right)

1. Incubate control
2. PBS- (Exposure control)
3. Nicotine 0.03 mg/mL
4. Nicotine 0.3 mg/mL

Membrane was cut horizontally at 75 kD molecular weight and slightly below 50 kD.

Blot image

Blot merged with ladder

ACE2 (~100 kD)

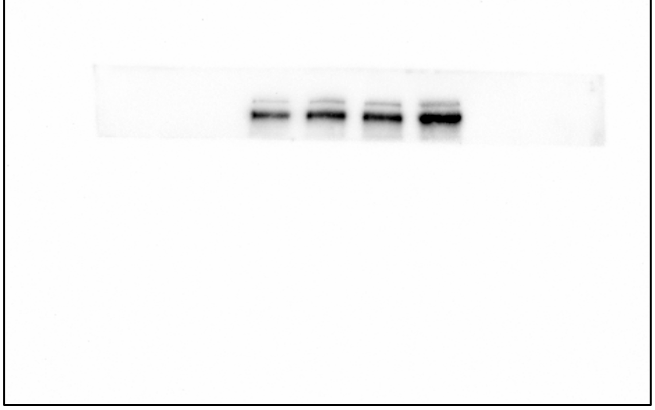

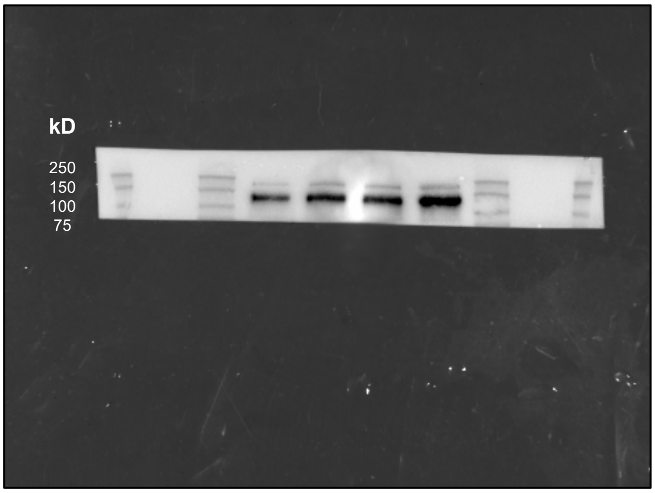

TMPRSS2 (~70 kD)

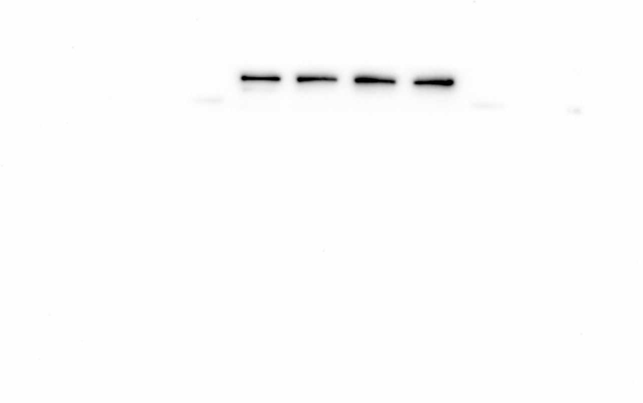

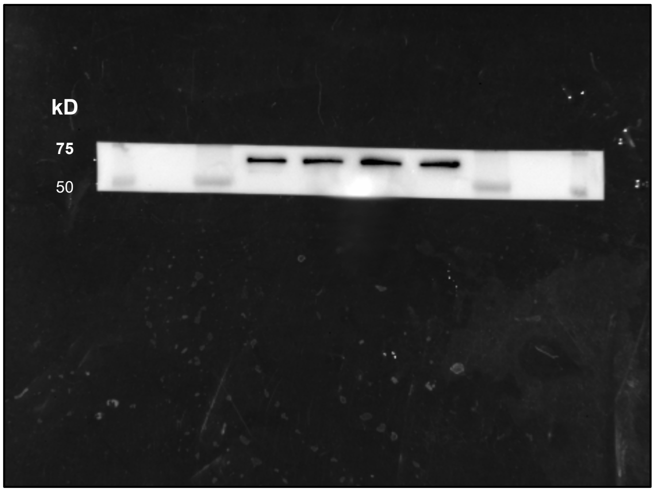

GAPDH (37 kD)

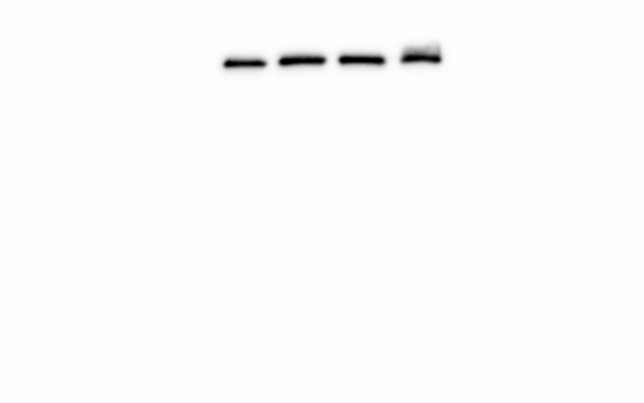

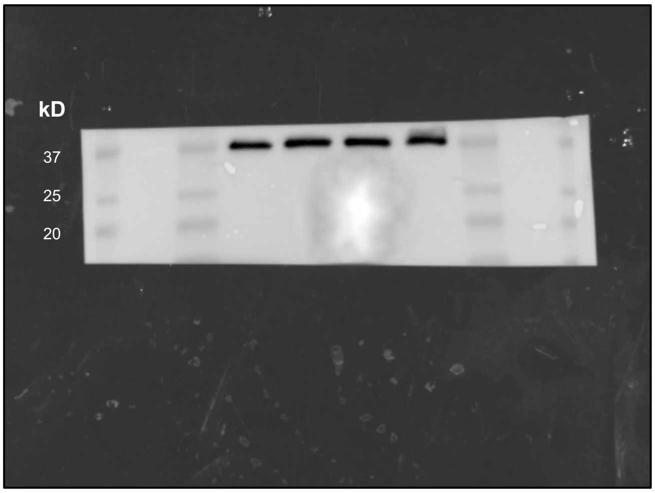

Cultex ALI Western blot 1
Membrane 1

Samples order (left to right)
1. Incubate control
2. Clean air (Exposure control)
3. PG/VG
4. PG/VG + Nicotine 6 mg/mL
5. PG/VG + Nicotine 60 mg/mL
6. JUUL

Membrane was cut horizontally at 75 kD molecular weight and slightly below 50 kD.

Blot image | Blot merged with ladder

ACE2 (~100 kD)

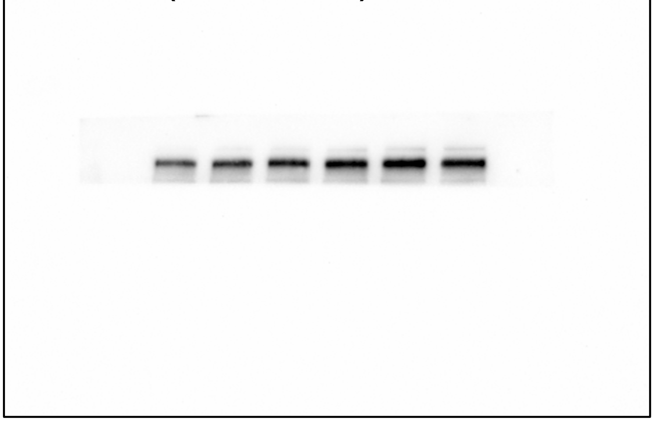

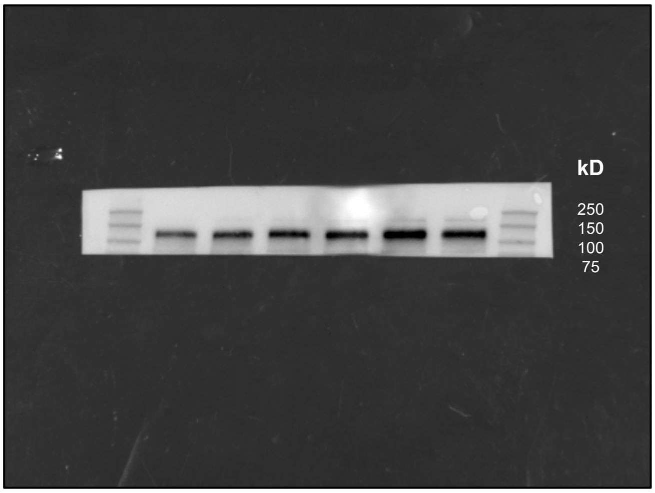

TMPRSS2 (~70 kD)

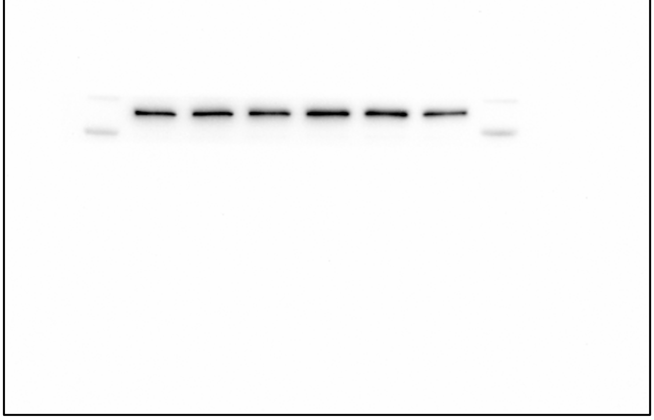

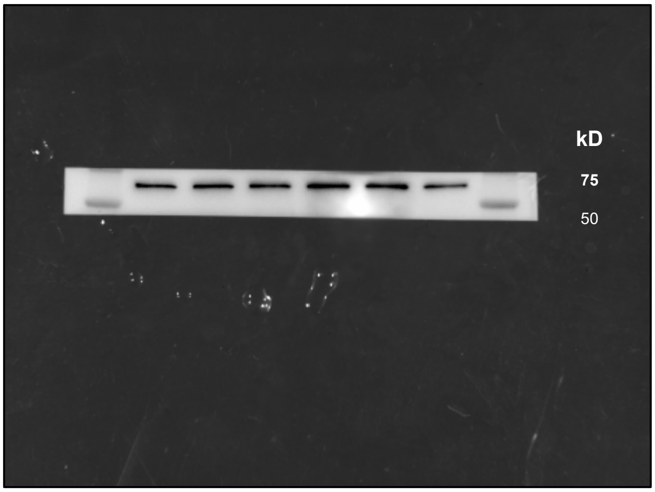

GAPDH (37 kD)

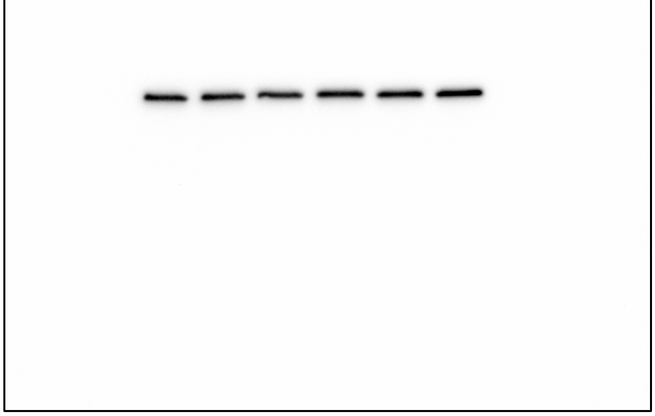

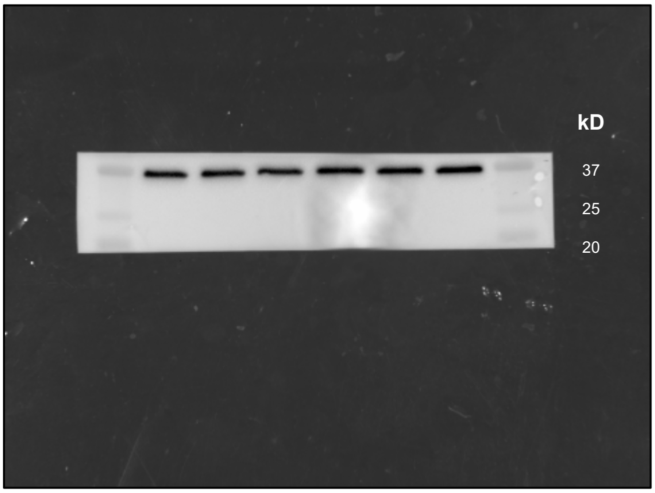

Cultex ALI Western blot 2
Membrane 2

Samples order (left to right)
1. Incubate control
2. Clean air (Exposure control)
3. PG/VG
4. PG/VG + Nicotine 6 mg/mL
5. PG/VG + Nicotine 60 mg/mL
6. JUUL

Membrane was cut horizontally at 75 kD molecular weight and slightly below 50 kD.

**Blot image**	**Blot merged with ladder**

ACE2 (~100 kD)

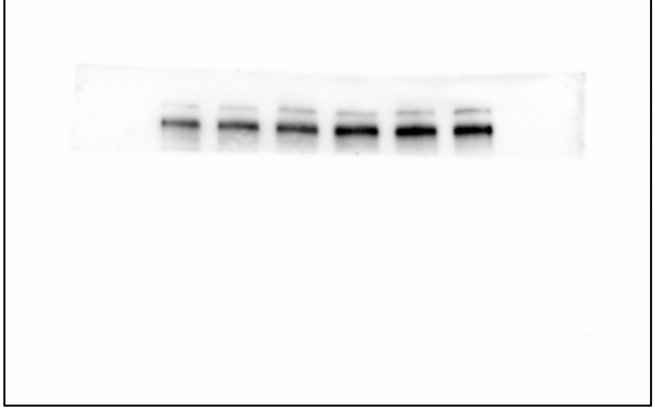

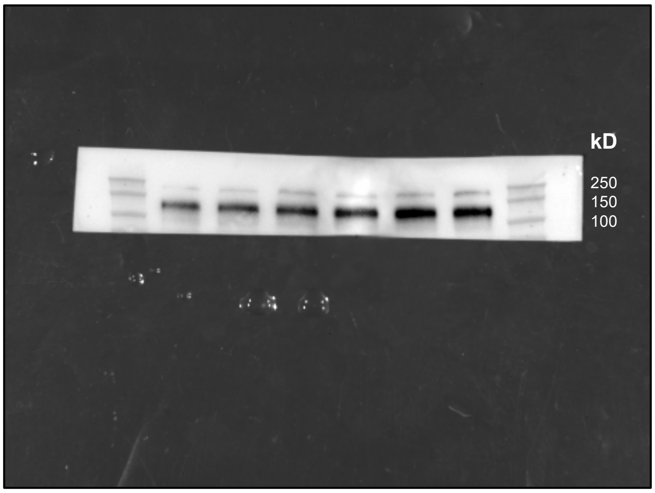

TMPRSS2 (~70 kD)

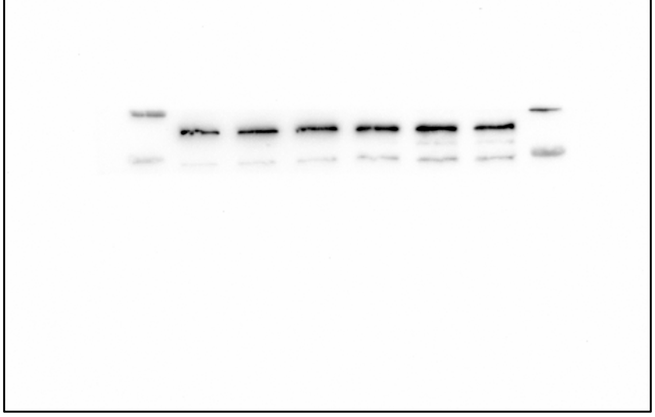

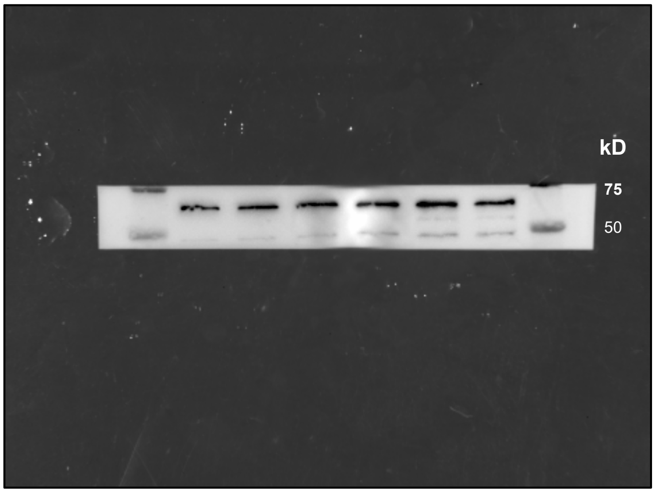

GAPDH (37 kD)

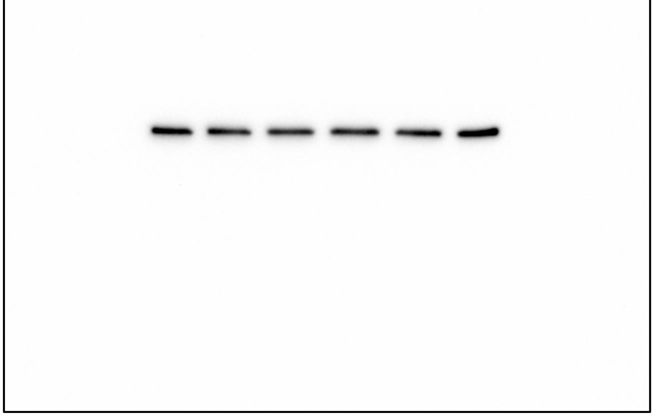

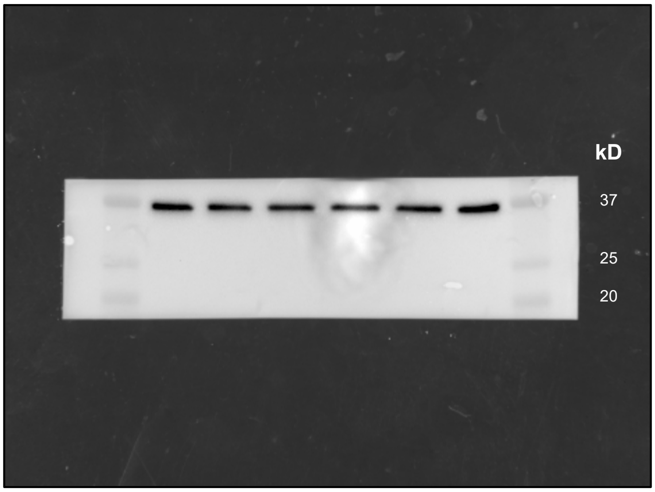

Cultex ALI Western blot 3
Membrane 3

Samples order (left to right)
1. Incubate control
2. Clean air (Exposure control)
3. PG/VG
4. PG/VG + Nicotine 6 mg/mL
5. PG/VG + Nicotine 60 mg/mL
6. JUUL

Membrane was cut horizontally at 75 kD molecular weight and slightly below 50 kD.

Blot image

Blot merged with ladder

ACE2 (~100 kD)

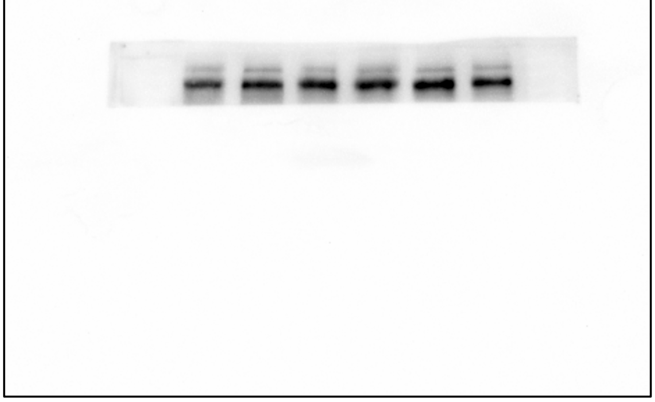

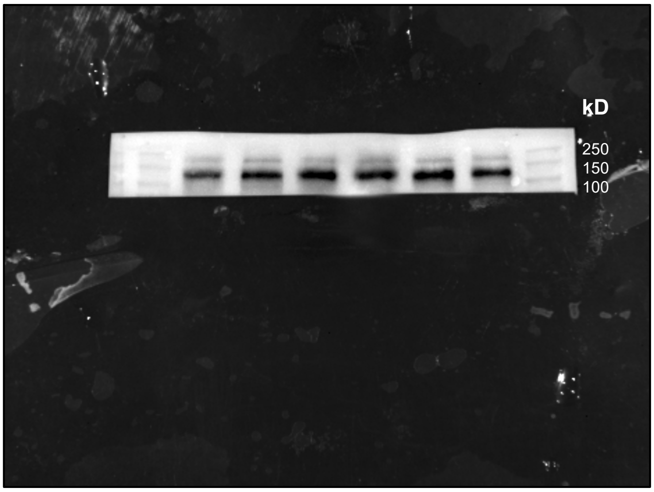

TMPRSS2 (~70 kD)

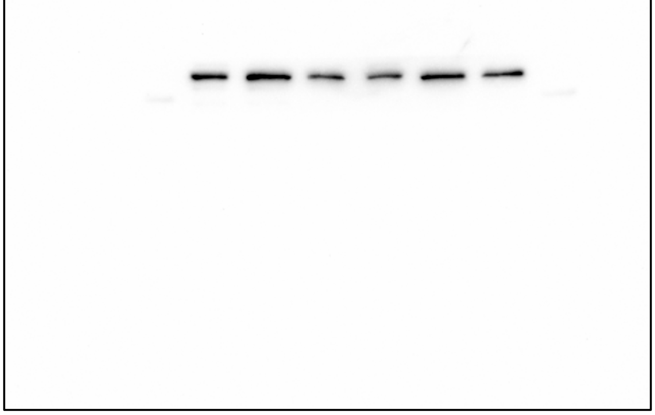

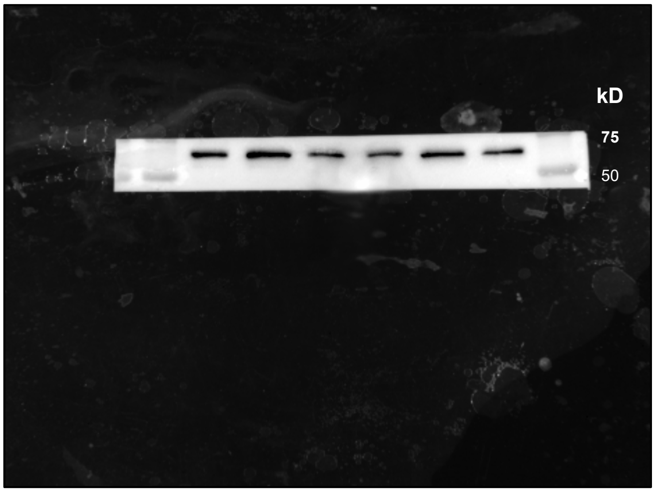

GAPDH (37 kD)

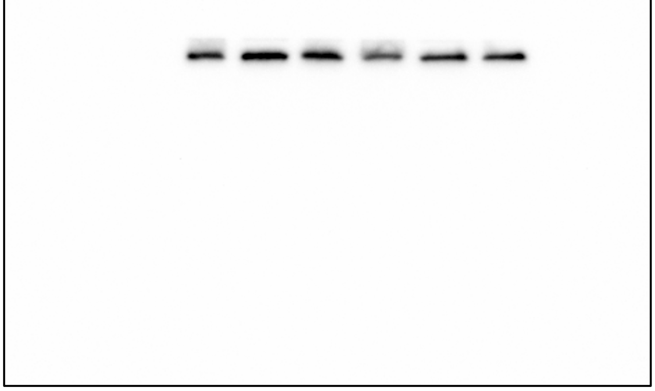

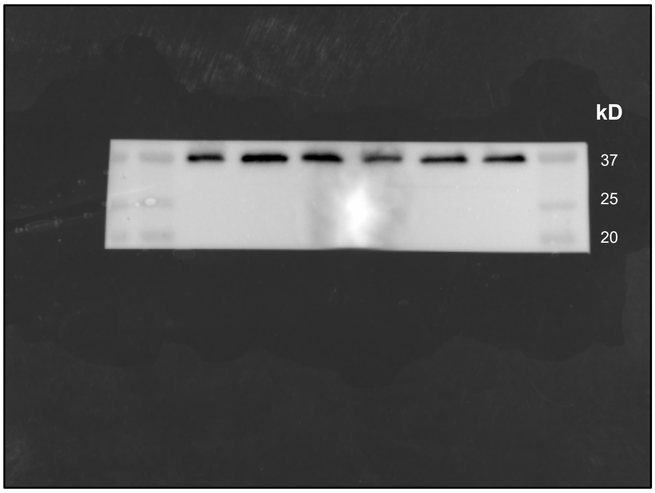

Supplement: Supplementary file 1 — Supplementary Information. [file 41598_2023_31592_MOESM1_ESM.pdf]
